# Intranasal delivery of mitochondria targeted neuroprotective compounds for traumatic brain injury: screening based on pharmacological and physiological properties

**DOI:** 10.1186/s12967-024-04908-2

**Published:** 2024-02-16

**Authors:** Jignesh D. Pandya, Sudeep Musyaju, Hiren R. Modi, Starlyn L. Okada-Rising, Zachary S. Bailey, Anke H. Scultetus, Deborah A. Shear

**Affiliations:** TBI Bioenergetics, Metabolism and Neurotherapeutics Program, Brain Trauma Neuroprotection (BTN) Branch, Center for Military Psychiatry and Neuroscience (CMPN), Walter Reed Army Institute of Research (WRAIR), 503 Robert Grant Avenue, Silver Spring, MD 20910 USA

**Keywords:** Traumatic brain injury, Intranasal drug delivery, Mitochondrial function therapeutics, Blood brain barrier, Neuroprotection

## Abstract

Targeting drugs to the mitochondrial level shows great promise for acute and chronic treatment of traumatic brain injury (TBI) in both military and civilian sectors. Perhaps the greatest obstacle to the successful delivery of drug therapies is the blood brain barrier (BBB). Intracerebroventricular and intraparenchymal routes may provide effective delivery of small and large molecule therapies for preclinical neuroprotection studies. However, clinically these delivery methods are invasive, and risk inadequate exposure to injured brain regions due to the rapid turnover of cerebral spinal fluid. The direct intranasal drug delivery approach to therapeutics holds great promise for the treatment of central nervous system (CNS) disorders, as this route is non-invasive, bypasses the BBB, enhances the bioavailability, facilitates drug dose reduction, and reduces adverse systemic effects. Using the intranasal method in animal models, researchers have successfully reduced stroke damage, reversed Alzheimer’s neurodegeneration, reduced anxiety, improved memory, and delivered neurotrophic factors and neural stem cells to the brain. Based on literature spanning the past several decades, this review aims to highlight the advantages of intranasal administration over conventional routes for TBI, and other CNS disorders. More specifically, we have identified and compiled a list of most relevant mitochondria-targeted neuroprotective compounds for intranasal administration based on their mechanisms of action and pharmacological properties. Further, this review also discusses key considerations when selecting and testing future mitochondria-targeted drugs given intranasally for TBI.

**Graphical Abstract:**

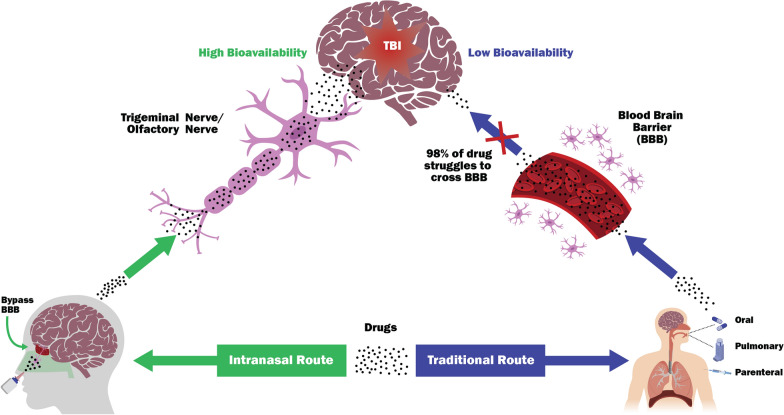

## Highlights

- Noninvasive intranasal drugs administration bypass the BBB, and can be rapidly delivered from the nasal mucosa to the brain
- The intranasal delivery is an attractive route for mitochondria-targeted neuroprotective drugs administration
- Accurate screening of intranasal compounds based on physiochemical properties is crucial
- When optimizing the intranasal administration by nanocarriers, drugs protection from chemical and enzymatic degradation must be carefully applied
- The intranasal route offers means to pharmacologically counter TBI pathogenesis in austere combat settings.

## Background

Traumatic brain injury (TBI) is one of the most common medical emergencies with consequences that worsens rapidly without immediate treatment [1, 2]. In the United States, approximately 4.8 million people are evaluated in emergency departments for TBI annually. An estimated 1.5 million Americans sustain a TBI each year, 230,000 hospitalizations, and about 50,000 deaths in the United States. For moderate to severe TBI patients, about 80,000–90,000 people experience the long-term disability [3]. In 2000, there were 10,958 TBI diagnoses. In 2015, this number jumped to 344,030 [4]. The incidence and prevalence of TBI rose globally in the past few decades.

Military service members are at high risk of TBI during combat missions. Because of the austere setting of the combat environment, the ideal treatment protocol, particularly for acute point-of-injury treatment, faces numerous constraints not encountered in civilian trauma centers. Therefore, researchers continue to explore therapeutic compounds with a neuroprotection potential that could be delivered immediately, and with ease to mitigate the progression of TBI pathogenesis [5]. Overall, neuroprotective compounds' clinical development is challenged by a consistent lack of clinical efficacy resulting in dozens of failed clinical trials over the past 3 decades [6].

Currently, no therapeutic intervention is available as neuroprotective treatment for TBI. In the battlefield, supportive measures usually include restoration of blood pressure and tissue oxygenation through resuscitation or control of intracranial hypertension with hypertonic saline. However, all these measures require skilled paramedics and reasonable medical settings, which are often not feasible during combat. Thus, there is a critical need to develop small, ruggedized devices and drugs that can either be self-administered or administered by non-medical personnel in the field. In this line of effort, US Army has tested nasal atomizer device in the emergency setting to administer analgesics, such as ketamine [7].

TBI is characterized by both the primary damage resulting from mechanical disruption of brain structures and the immediate emergence of secondary pathogenic molecular events, which collectively contribute to neurological deficits. The secondary (i.e., acute, sub-acute and chronic) effects are defined as processes experienced within days, up to several weeks post-injury [8–10]. Much of our understanding of the pathobiology of TBI have arisen from animal models that mimic features of human TBI. There are several detailed reports on models and cellular mechanisms of TBI [8, 11, 12].

Importantly, mitochondrial dysfunction is a shared immediate common indicator of cellular damage for multiple preclinical TBI models, including penetrating traumatic brain injury (PTBI), controlled cortical impact (CCI), blast (BTBI), and closed head injury (CHI) [13–17]. Mitochondrion is probably the most studied subcellular compartment due to its indispensable role in the regulation of cellular homeostasis and multifaceted functions. At the cellular level, the main deleterious effects of the secondary TBI cascades are cell damage and death, that are centrally regulated by mitochondria. Excitotoxicity, calcium overload and membrane permeability transition, metabolic and bioenergetic failure, antioxidants depletion, free radicals over production and oxidative stress, elevated calpains, caspases, and apoptosis inducing factors are key mechanisms governing mitochondria-mediated neuronal damage following TBI [18, 19]. Thus, mitochondrial dysfunction disrupts cellular homeostasis, exacerbating the acute through chronic progression of TBI pathogenesis. Mitochondria have become a major pharmacological target in TBI and many neurodegenerative diseases due to governing vital cellular functions and cell death [15, 19–21]. In theory, and as reported in many preclinical studies, the secondary TBI pathogenesis should be amenable to mitochondria-targeted pharmacological interventions resulting in improved outcomes [22]. Strikingly, despite the promising preclinical results of the neuroprotective efficacy of mitochondria-targeted drugs, these compounds have failed to translate successfully to clinical studies.

One of the greatest obstacles to the successful delivery of drug therapies to the central nervous system (CNS) is the blood brain barrier (BBB). Although a restricted class of lipid-soluble drugs (< 400 Dalton) cross freely, the BBB prevents 98% of small and 100% of large molecules from entering the brain [23, 24]. Moreover, even small molecule (< 400 Dalton) drugs must meet certain criteria in order to effectively cross the BBB (i.e., nonpolar and not multi-cyclic) [25]. Many neuroprotective compounds do not have these properties, imparting severe restraint to the progress of TBI treatment development. At the application level, 98% of drug compounds do not cross the BBB in therapeutic quantities [26]. According to the US Food and Drug Administration (FDA), more than 90% of neuroprotective drugs tested at the clinical level to treat central nervous system (CNS) diseases have not been approved due to their poor bioavailability [27]. Thus, alternative routes of drug administration to the traditional parental or oral route, especially one that circumvents the multitude of barriers inhibiting brain penetration by neuroprotective compounds may solve this problem.

The most widely tested drug administration routes for TBI at the preclinical level are parental (e.g., intravenous, IV; intramuscular, IM; and intraperitoneal, IP) and oral (e.g., intragastric, IG) routes. These standard systemic routes of drug delivery typically require higher levels of dosing to reach targeted CNS concentration, often increasing the risk of adverse effects and toxicity while failing to achieve the desired therapeutic efficacy. Importantly, some drugs such as proteins and peptides are inappropriate for oral administration because they are significantly degraded by gastric acid and proteolytic enzymes in the gastrointestinal (GI) tract, and rapidly metabolized by the first-pass effect in the liver. Following IP administration, drugs are absorbed into the mesenteric vessels, which drain into the portal vein and pass through the liver. In the liver, a portion of the drug gets metabolized and significantly excreted, decreasing the bioactive drug concentration before it reaches systemic circulation [28]. Even drugs administration using IM and IV routes are challenged by first-pass liver metabolism. Intracerebroventricular (ICV) and/or intraparenchymal routes may provide effective delivery of small and large therapeutic molecules, including beneficial growth factors for preclinical neuroprotection studies. However, clinically these delivery methods are invasive and risk inadequate CNS exposure due to the rapid turnover of cerebral spinal fluid (CSF). Furthermore, treatment development in TBI is also complicated by the effects of TBI on the pharmacokinetics of drugs. TBI-induced increased hepatic metabolism and decreased plasma protein binding resulted in higher drug clearance and decreased bioavailability of potential neuroprotective therapies [29].

**Fig. 1:**
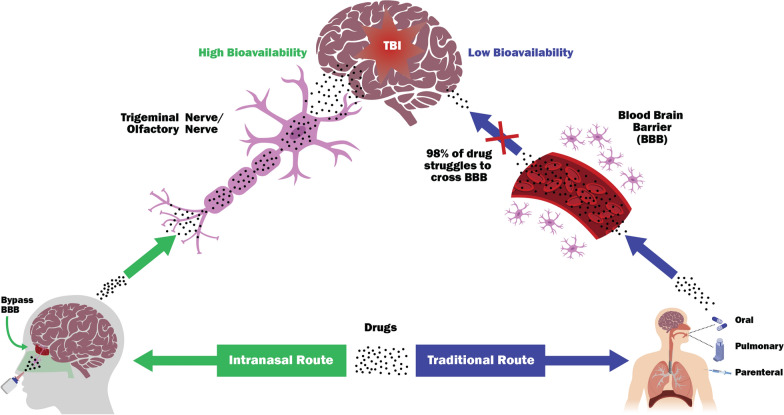
Schematic representation of key aspects of intranasal delivery of neuroprotection compounds to the brain. TBI is difficult to treat as most therapeutic agents (98%) cannot reach in the brain, mainly due to the selective permeability of the blood–brain barrier (BBB). The olfactory and trigeminal nerves can serve as direct nose-to-brain routes that bypass the BBB that can impede absorption of most CNS targeted compounds, resulting in higher bioavailability. In addition, compared to traditional routes, the nasal administration of drugs can direct the rapid CNS absorption to brain tissues, thereby circumventing the hepatic first-pass metabolism and gastric degradation and allowing fast onset of pharmacological action

The direct intranasal drug delivery approach to therapeutics, first developed and patented by William H. Frey in 1989 [30, 31] represents a non-invasive method for bypassing the BBB via the olfactory route. The graphical abstract illustrates the concept of higher bioavailability of drugs achieved at the brain target when administered through intranasal route following TBI (Fig. 1). This route of administration has the added benefit of being able to achieve much higher concentrations in the injured brain in the absence incurring adverse systemic effects. Administration of radio-labeled proteins, such as insulin-like growth factor-1 (IGF-1) and interferon-beta 1b (IFN-β1b), into the rat nose leads to their distribution along trigeminal and olfactory nerve associated pathways and dissemination into both rostral and caudal regions of the rat brain within 30–60 min, thus providing evidence of rapid brain access of CNS acting drugs through intranasal delivery [32, 33]. Using the intranasal method in animal models, researchers have successfully reduced stroke damage, reversed Alzheimer's neurodegeneration, reduced anxiety, improved memory, and delivered neurotrophic factors and neural stem cells to the brain [30, 31, 34–37].

Intranasal administration may be particularly relevant for military combatants as a TBI point-of-injury solution in the battlefield because over 80% of military-centric TBIs result from blast and/or impact concussion. Some major advantages of the intranasal drug delivery method include: (1) the absence of GI tract-associated drug degradation; (2) the hepatic first-pass metabolism is bypassed, thus increasing drug bioavailability; (3) while nasal bioavailability for smaller drug molecules is advantageous; absorption enhancers can further boost bioavailability of compounds as needed; (4) the anatomy of the nasal region provides a direct path to the CNS that bypasses the BBB as the drug gets rapidly absorbed via the highly-vascularized nasal mucosa; (5) offers alternate routes for rapid medication and drug delivery by non-medical personnel or self-administration, when IV access is unavailable; and (6) there is a low potential for injuries or blood-borne disease transmission compared to parental routes. Thus, it is not surprising that intranasal administration enables drugs to directly access the brain with additional benefits compared to traditional routes of administration.

While there are still some limitations with intranasal delivery, including solubility, pH, and dose/volume limits, it is becoming increasingly accepted that this route is both safe and effective [38]. A meta-analysis of the subjective reactions, safety, and side-effects to intranasal delivery of oxytocin, steroids, insulin, and benzodiazepines revealed no significant adverse side-effects [39–42]. Intranasal drug delivery method should be considered as a viable drug delivery route in TBI that has enormous clinical implications for achieving more robust efficacy in the injured brain while mitigating potentially adverse systemic effects.

Under the umbrella of neurotherapeutics development for military medicine, we aim to deliver mitochondrial targeted drugs at varied concentrations to the injured brain. Since mitochondria are the core mediator of the secondary injury cascades in TBI and serve as an important target in preventing neuronal cell death, this review examines previous and ongoing studies exploring intranasal routes delivery of mitochondrial drugs used in CNS diseases and TBI. Our focus remains on detailing the mechanisms of action and pharmacological profile of each identified compound tested intranasally. Additionally, the prospects and challenges/limitations of the intranasal route of drug delivery for TBI are also discussed.

### Blood-CNS barriers (BCB) and TBI

**Fig. 2:**
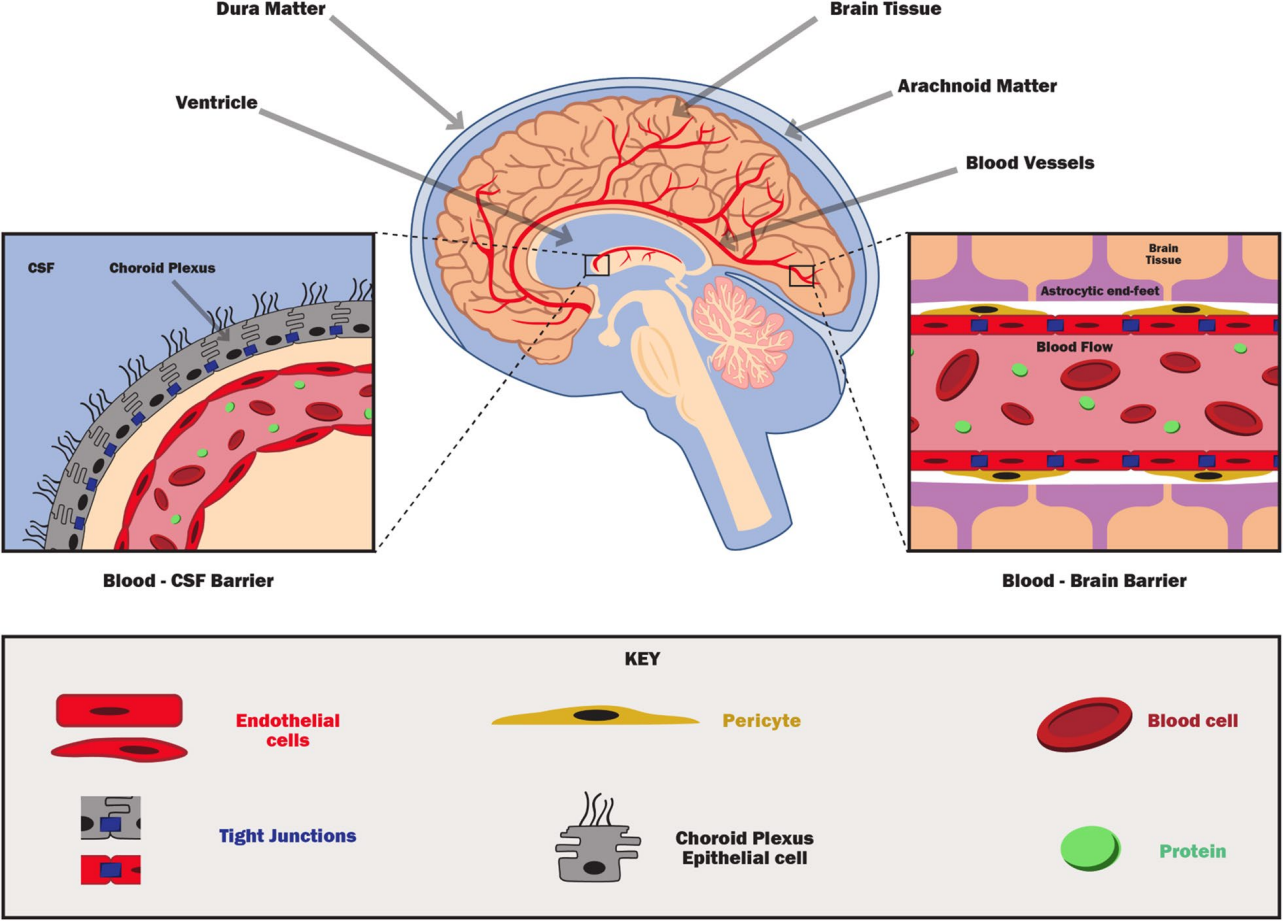
Comparison between the Blood-CSF Barrier (BCSFB) and Blood–Brain Barrier (BBB) structures. The BBB separates the lumen of the brain capillaries from the brain parenchyma. The main contribution to the BBB property of reduced permeability comes from the tight junctions (TJs) among endothelial cells lining of the capillaries. The BCSFB is at each ventricle's choroid plexus epithelial cells, joined together by TJs. Unlike the endothelium in the brain parenchyma, capillaries of the choroid plexus have no TJs and are fenestrated; therefore, they are relatively leaky and permeable to small molecules. Although in principle, both the barriers serve the homologous defensive purpose for the CNS, their distinct anatomical feature allows the interchange of different substances between the CSF/bloodstream and the brain cells

Before discussing the intranasal compounds, here we introduce the concept of physiological blood-CNS barriers (BCB), and explore the latest discovery regarding the effects of TBI pathology on these barriers, and introduce various therapies that would benefit the BCB integrity. The CNS compartments are tightly sealed from the changeable milieu of blood by the BBB as well as the blood–CSF barrier (BCSFB). BBB and BCSFB together form the anatomical BCB to shield brain against potentially toxic substances. While the BBB is localized at the level of the endothelial cells within CNS blood vessels, the BCSFB is formed by choroid plexus epithelial cells (Fig. 2). The BBB permits exchange of gases, amino acids, and metabolites like glucose, but inhibits the diffusion of water-soluble molecules by a network of tight junctions (TJs) that interconnect the endothelial cells, in conjunction with the absence of fenestrae. The BBB allows the transport of biomolecules (≤ 400 Daltons) directly to the brain cells without the involvement of CSF [23]. Whereas the BCSFB is at the epithelial cells of choroid plexus, which are joined by TJs. The capillaries in the choroid plexus differ from BBB, as molecules of larger size may be able to freely move across the endothelial cells to CSF through fenestrations and intercellular gaps. This diffusion of molecules facilitates the exchange of metabolites between CSF and blood. Since, no diffusional barrier exists in between CSF and nervous tissues’ interstitial space, even larger size may be able to enter up to the interstitial space by diffusion in the vicinity of CSF existence, but may not be able to be taken up by neuronal cells [43]. Drug entry into neuronal cells is dependent on individual physiochemical properties. Therefore, penetration of drug up to interstitial space is expected through BCSFB at a rate inversely related to the molecular weight / size. However, it does not provide information on rates of neuronal drug uptake across the BBB at the brain capillary endothelium level [23]. Neurotherapeutics entry into CSF is reported for many CNS diseases, however it does not provide information if drug may readily cross the BBB, and reach to the neuronal target. [23].

Overall, the entry of drugs into the CNS depends on the pharmacokinetics (PK) parameters of drugs, such as molecular size, electric charge, lipophilicity, plasma protein binding, affinity to diffusion/active transport systems at the BBB and BCSFB, and pharmacodynamic (PD) factors such as CSF flow in the brain. Knowledge of the PK-PD profile of neuroprotective drugs at the BBB/BCSFB level may be helpful to improve the therapeutic window of opportunity to treat TBI and other CNS diseases. The ideal therapeutic compound for TBI and CNS diseases should have PK-PD criteria such as smaller molecular weight, moderately lipophilic, low plasma protein binding affinity, higher distribution volume, and serve as weak ligand of P-glycoprotein or another efflux pump located at the BCB [44]. When several approximately equally active compounds are available, a drug that comes closer to these physicochemical and pharmacological (PK-PD) properties should be preferred.

Notably, BCBs are the critical mediator and modulator of TBI pathology progression. Following TBI, direct or indirect mechanical forces to the brain causes vascular and parenchymal damage contributing to BBB/BCSFB breakdown. Subsequently, this may affect the physicochemical and pharmacological responses of neuroprotective drugs bioavailability in the injury regions during acute to sub-acute TBI conditions. In recent years, the pathophysiology of the BBB breakdown and its downstream effects, such as edema, inflammation, ischemia and hyperexcitability has become increasingly evident [45]. TBI disrupts expression of TJs proteins altering dynamics of BCB [45]. Additionally, studies have shown that TBI promotes BCB opening as early as the day after injury, and BCB can remain open up to 30 days [46, 47].

The ideal neuroprotection strategy would be to prevent BCB breakdown and stabilize it, thereby protecting the brain from factors released from damaged blood vessels that cause further damage. Therefore, attempts have been made at curtailing the permeability of BCB. Studies have shown that blocking VEGF, a promoter of angiogenesis, decreases BBB permeability in vivo by reducing permeable micro-vessel formation [18]. Numerous hormones including neural growth factors, ghrelin, and progesterone have recently been found to have neuroprotective effects following TBI, and they influence the BBB integrity [48–50]. More importantly, an innovative study suggests that mesenchymal stem cells and fibroblast growth factor 21 may mitigate BBB breakdown following TBI [51, 52]. For instance, endothelial mitochondria have been recognized as a key player in BBB permeability and maintaining their function as a potential new therapeutic strategy [53]. Thus, targeting mitochondria and endothelial cell mitochondrial regulation is potential new therapeutic strategy to maintain BBB integrity. Although BCB disruption is a pathological hallmark of TBI, further elucidation of the dynamics of BBB/BCSFB dysfunction after TBI would provide important information for validation of drug selection based on the optimal therapeutic dose and window of opportunity, and best route of administration in preclinical TBI models.

In contrast, transient disruption of the BBB to increase the concentration of neurotherapeutics has been explored. Invasive methods that primarily rely on disruption of the BBB integrity by osmotic or biochemical means, or direct intracranial drug delivery by intracerebroventricular, intracerebral or intrathecal administration after creating reversible openings in the head are recognized [54]. However, safety and toxicity challenges associated with these techniques limit their application. Therefore, safe method through intranasal route that can enhance drug delivery to the CNS are of great pharmaceutical interest.

## Method

### Selection criteria

We searched research articles exclusively published in peer-reviewed journals. Additionally, potentially eligible articles were also obtained by the Google Scholar website (https://scholar.google.com). The keywords used in the literature search were "Intranasal drug AND Mitochondria AND Traumatic Brain Injury AND CNS Disease" in the reporting period between 1989 and 2023. Approximately 18200 articles were identified based on keyword search. After filtering through the layers of inclusion criteria, we selected 24 compounds, the most pertinent drug candidates listed in Table 1. Article screening was conducted initially using abstracts followed by full-text level.

**Table 1:** List of potential mitochondria-targeted intranasal compounds used in preclinical studies

Compound name	Compound class	Chemical properties	Pharmacokinetic information	Tested CNS diseases	Mechanism of action (in relation to mitochondria)
Nicotinamide mononucleotide (NMN)/Nicotinamide adenine Dinucleotide (NAD) [58]	Empirical Formula: C_21_H_28_N_7_O_14_P_2_ Therapeutic: Cofactor/Coenzyme	PH: Base logP: 6.38 pKa: -1.2 (Predicted)	Delicate molecule, oral consumption may destroy it NAD half-life 1- 2 h	Transient Focal Ischemia, TBI, Neurodegenerative diseases	Restores mitochondrial function via participation TCA cycle and oxidative phosphorylation
N-acetylcysteine amide (NACA) [59]	Empirical Formula: C_5_H_10_N_2_O_2_S, amide of NAC Therapeutic: Antioxidant, Anti-inflammatory	PH: Base logP: -0.44 pKa: 9.52	BBB permeant, half‐life 6.25 h [60]	TBI, Acute Cognitive Dysfunction, PD	Reduces oxidative stress, improves mitochondrial bioenergetics, and maintains mitochondrial glutathione content
SKQ1/Mito Q [61]	Empirical Formula: C_36_H_42_BrO_2_P/C_37_H_44_O_4_P Therapeutic: Antioxidant	PH: logP: pKa:	Bioavailability limited by intracellular metabolism [62]	TBI, AD PD	Selectively blocks mitochondrial oxidative damage and prevents cell death
Curcumin [63–67]	Empirical Formula: C_21_H_20_O_6_ Therapeutic: Anti-inflammatory, Anti-tumor, Antioxidant	PH: Neutral logP: 3.62 pKa: 9.06	Poor oral absorption, IN delivery enhanced brain-uptake efficiency	AD, Brain Inflammation [68], Dementia	Protects mitochondria from oxidative damage and attenuates apoptosis
Resveratrol [69, 70]	Empirical Formula: C_21_H_20_O_6_, polyphenolic phytoalexin Therapeutic: Antioxidant	PH: Neutral logP: 2.57 pKa: 8.49	High oral absorption, but low bioavailability [71]	AD, Autism, TBI, Brain Ischemia [72]	Induces mitochondrial biogenesis through SIRT pathway [72]
Rivastigmine [73, 74]	Empirical Formula: C_14_H_22_N_2_O_2_, carbamate ester Therapeutic: Cholinergic agent	PH: Acid logP: 2.45 pKa: 8.89	Half-life 1.5 h, and volume distribution—1.8–2.7 L/kg [75]	AD, Dementia, PD ETC [76]	Enhances mitochondrial ETC function, increases enzymatic activities of diverse complexes and oxidative capacity of the ETC [76]
Cyclin D1 [77]	Empirical Formula: C_43_H_71_N_9_O_11_S_2_ Therapeutic: Cell cycle regulator	PH: Acid logP: -0.3 pKa:	Half-life < 30 min [78]	TBI	Regulates mitochondrial function by coordinating metabolic substrate utilization within the cell [77]
Pitavastatin [79, 80]	Empirical Formula: C_23_H_36_O_7_ Therapeutic: HMG-CoA Reductase Inhibitor	PH: Acid logP: 3.5 pKa: 4.3	Water Soluble, half-life ~ 12 h [81]	TBI, AD [82]	Attenuates AGEs-induced mitophagy via inhibition of ROS generation in the mitochondria [83]
Caspase-1 Inhibitor Boc-D-CMK [84]	Empirical Formula: C_17_H_22_ClNO_5_ Therapeutic: Caspase-1 Inhibitor	PH: logP: 2.7 pKa:		AD, Multiple Sclerosis, Neurodegenerative diseases [85]	Decreases mitochondrial dysfunction, and attenuates caspase-3-dependent apoptotic pathway [86]
Pentoxifylline [87]	Empirical Formula: C_13_H_18_N_4_O_3_, a xanthine derivative Therapeutic: Phosphodiesterase inhibitor (Vasoactive)	PH: Base logP: 0.3 pKa: 19.64	Parental compound half-life- 0.4–0.8 h, and it’s metabolites half-life- 1–1.6 h [88]	It affects the Nrf2 antioxidant response elements pathway; thus, could help treat TBI or AD [89]	Reverses oxidative mitochondrial defect in claudicating skeletal muscle [90]
Ketamine [91]	Chemical: C_13_H_16_ClNO Therapeutic: Anesthesia	PH: Acid logP: 4.7 pKa: 2.2	Distribution half-life is about 7 -11 min, and the elimination half-life is about 2.5–3 h [92]	TBI [93]	Ketamine restores levels of malondialdehyde, glutathione peroxidase and superoxide dismutase
Tetrandrine [94]	Chemical: C_38_H_42_N_2_O_6_, a bis-benzylisoquinoline alkaloid Therapeutic: Calcium Channel Inhibitor	PH: Base logP: 5.55 pKa: 8.28	Very low bioavailability, which is why intranasal delivery is an essential route	Ischemic Stroke, TBI [94]	Prevents doxorubicin-induced mitochondrial impairment [95]
Insulin [96]	Empirical Formula: C_257_H_383_N_65_O_77_S_6_ Therapeutic: Peptide hormone	PH: logP: pKa:	Half-life 4–6 min	TBI, AD, PD, Neuronal Apoptosis [97, 98]	Increases expression of mitochondrial proteins, oxidative enzyme activity and ATP synthesis in muscle [97, 99, 100]
Geraniol [101]	Empirical Formula: C_10_H_18_O is a monoterpene Therapeutic: Anti-inflammatory, antioxidant	PH: Neutral logP: 2.89 pKa:16.33	BBB permeable, Half-life 12 min [102]	Cerebral Ischemia, PD, Neurological diseases [103]	Increases cell viability, preserved mitochondria membrane potential and improves the level of mitochondrial complex 1 [104]
Muscone [105]	Empirical Formula: C_16_H_30_O Therapeutic: Neuroprotectant	PH: logP: pKa:	BBB permeable [106], IN delivery serves as a route for rapid drug entry into the brain [107]	TBI, Ischemia injury Spinal Cord Compression [106]	Regulating Drp1‐dependent mitochondrial fission [106, 107]
Davunetide [108, 109]	Empirical Formula: C_36_H_60_N_10_O_12_ Therapeutic: growth factor	PH: Acid logP: -2.6 pKa: 3. 33 [110, 111]	Well tolerated after IN or IV administration [112]	AD, Stroke, Hypoxia [111]	Inhibition of programmed cell death and correction of mitochondrial dysfunction
Apelin-13 [113]	Empirical Formula: C_69_H_111_N_23_O_16_S Therapeutic: Anti-inflammatory [113]	PH: logP: pKa:	Half-life 5–8 min [113]	Brain Ischemia [113]	Prevented serum deprivation (SD)-induced mitochondrial depolarization and apoptotic events [114]
Quercetin [115]	Empirical Formula: C_15_H_10_O_7_ is a polyphenolic flavonoid Therapeutic: Anti-inflammatory, Neuroprotectant	PH: Neutral logP: 1.81 pKa:6.44	Poor solubility, poor oral absorption	AD, Brain Ischemia	Modulates pathways associated with mitochondrial biogenesis and mitochondrial membrane potential
DL-3-n-butylphthalide (NBP) [116, 117]	Empirical Formula: C_12_H_14_O_2_ Therapeutic: Nutraceuticals, Antioxidants	PH: Weak Base logP: 3 pKa: 14.18	Fat-soluble, half-life 11.84 h	Ischemic Stroke, TBI, Neurodegenerative diseases	Prevents Oxidative damage and reduces mitochondrial dysfunction
Gallotannin [118]	Empirical Formula C_27_H_24_O_18_ is a polyphenolic compound Therapeutic: PARG inhibitor	PH: Acid logP: 4.73 pKa: 7.61	Poor bioavailability, large size, high affinity to plasma proteins	Neurodegenerative diseases, Brain Ischemia [118]	PARP inhibition protects mitochondria and reduces ROS production [118]
Progesterone [119]	Empirical Formula: C_21_H_30_O_2_ is a steroid hormone Therapeutic: Endogenous sex hormone	PH: Base logP: 3.58 pKa:18.92	Low oral bioavailability, half-life is 5–20 min [120]	Stroke, TBI, Spinal Cord Trauma, Central and peripheral neuropathies	Stimulates mitochondrial biogenesis and enhances mitochondrial functional efficiency and increased metabolic rates [121]
Huperzine A [122, 123]	Empirical Formula: C_15_H_18_N_2_O, sesquiterpene alkaloid Therapeutic: Acetylcholinesterase Inhibitor	PH: Base logP: 0.833 pKa: 7.7	Crosses BBB, half-life 10–14 h [124]	AD, Epilepsy, TBI	Attenuates apoptosis by inhibiting the mitochondria–caspase pathway directly and indirectly [125]
Ginsenoside Rg3 (GRg3) [126]	Empirical Formula: C_42_H_72_O_13_ Therapeutic: Natural steroid glycosides	PH: Base logP: 2.27 pKa: 12.9	Half-life 16 min [127]	Neurodegenerative disease, TBI, Brain Ischemia	Antioxidant, triggers mitochondrial rejuvenation

Reasons for inclusions and exclusions eligibility criteria were predetermined. A study was considered eligible if it tested a chemical compound with mitochondrial enhancing function as a mechanism of action for treating TBI or any CNS diseases. Mitochondria-targeted compounds, those under investigation for intranasal delivery and awaiting successful outcomes, also made into our short-list. Most of these compounds are antioxidants tested in several CNS diseases, including neurodegenerative diseases, TBI, stroke, multiple sclerosis, autism, and dementia. Other approaches were excluded from the current analysis, such as intranasal therapeutic device development, intranasal stem cell transplantation, and intranasal growth factors administration.

In the literature search, the selected drug candidates were used in preclinical or early clinical stages, and their successful claim related to intranasal administration of mitochondria-targeted therapeutics for TBI, or neurodegenerative diseases were limited to animal models only. To confirm their role in clinical research, we searched the Clinical Trial Database (https://clinicaltrials.gov) using the keyword "Intranasal Drug AND Mitochondria AND Brain Injury AND CNS Disease", which revealed zero results, confirming that none of these compounds have been tested clinically employing the intranasal route of drug delivery.

### Drug’s favorable properties for intranasal delivery

The selected intranasal deliverable drug candidates should be readily dissolved in the vehicle solvent, permeable to the nasal mucosa and meet clinical criteria for safe delivery. Drugs with lower molecular weight and higher lipophilicity (log P) generally favor rapid intranasal uptake and brain delivery. Additional critical pharmacological factors that dictate the bioavailability and efficacy of intranasal compounds include drug metabolism in the nasal cavity, degree of dissociation (pKa), chemical structure, drug half-life (t_½_), osmolarity and pH. The peptidase and protease activity can hinder the delivery of peptides and proteins in the nasal mucosa [55].

Ideal intranasal compounds should be able to overcome the enzymatic barrier created by these metabolic enzymes in the nasal epithelium. The pKa of a drug influences solubility, lipophilicity, protein binding and permeability and is of paramount importance to the overall characteristics of a drug [56]. Drugs that have a shorter half-life tend to act very quickly, but their effects wear off rapidly, meaning that they usually need to be administered several times a day intranasally to have the therapeutic effect. In contrast, a longer half-life requires less frequent dosing required, thus steady-state concentrations are more attainable, and therapies are more likely to be efficacious.

Studies have shown that hypotonic formulations improve drug permeability through the nasal mucosa [57]. The physiologic pH of the nasal mucosa is 5.0–7.0, and compounds with pH outside of this physiologic range may cause irritation to the nasal mucosa and may affect its absorption or may show adverse effects.

Based on these efficacy factors, we have illustrated a brief profile of all eligible intranasally tested mitochondria targeted therapeutics. We utilized PubChem (https://pubchem.ncbi.nlm.nih.gov) as the search tool to obtain these compounds' physiochemical properties.

### Evaluation of mitochondria targeting intranasal compounds

To compile the list of intranasal compounds in Table 1, we designed and used a structured data abstraction format to ensure consistency in appraising each intranasal drug. The most pertinent mitochondria targeting intranasal compounds from this table are selected for further discussion.

## Potential compounds

Numerous preclinical studies testing mitochondria targeted compounds have indicated that the direct delivery of mitochondrial drugs to the brain is achievable through the intranasal route (Table 1). However, the therapeutic efficacy testing of these preclinical drugs administered via intranasal delivery in humans remains to be elucidated. To translate preclinical success into clinical practice, the intranasal compounds with an established mechanism of action in preclinical studies should have several desirable characteristics, as listed above, and suggested by a physicochemical/pharmacokinetic literature survey. Besides well-established mechanisms that enhance mitochondrial function, we have considered desirable physicochemical properties in selecting the compounds for discussion.

### NMN & NAD

The NMN (nicotinamide mononucleotide) is a precursor of coenzyme nicotinamide adenine dinucleotide (NAD), which is a central coenzyme of redox reactions that restores mitochondrial function. NMN is a neutral compound, and it is lipophilic in nature. NAD also serves as a cofactor for enzymatic reactions to enhance energy metabolism via participation in pyruvate dehydrogenase, tricarboxylic acid cycle, and oxidative phosphorylation. Intracellular NAD has a short half-life estimated to be 1 to 2 h [128]; therefore, intranasal administration is preferable over systemic routes. A preclinical study revealed that intranasal administration with NAD profoundly decreased brain injury in a rodent model of transient focal ischemia [58]. In contrast, intravenous injection of identical dose of NAD could not produce significant improvement in ischemic brain injury. These results provide the first in vivo evidence that intranasal NAD administration may be a novel strategy for decreasing brain damage in cerebral ischemia. Another study reported that NMN attenuates brain injury after intracerebral hemorrhage by suppressing neuroinflammation/oxidative stress [129].

### NACA

The antioxidant NACA (N-acetyl cysteine amide), a glutathione (GSH) prodrug, reduces oxidative stress, improves mitochondrial bioenergetics, and maintains antioxidant capacity. It is a neutral, lipophilic compound with higher membrane permeability. The parental compound NAC (N-acetylcysteine) is undeniably effective in hepatotoxicity, particularly due to its glutathione replenishing and antioxidants effects. However, NAC is acidic in nature, and being a charged molecule, it has poor bioavailability in the brain due to BBB inhibition [130]. Conversely, NACA is a neutral compound with higher bioavailability due to the reactive amide group added to the parent compound.

Our recently conducted preclinical studies in the penetrating TBI animal model, demonstrated zero toxic effects for IP-administered NACA at the highest concentration (600 mg/kg), supporting a safer profile for its use as a mitochondrial-targeted neuroprotection compound for TBI (unpublished data). During the clinical trial of Parkinson’s disease (PD), oral NAC did not show significant increases in brain GSH, which may be related to its low bioavailability [131]. However, intranasal administration of NACA may rapidly achieve therapeutic concentrations in the brain. Indeed, the recent efficacy study of a nasal spray containing NAC in hypertonic solution for the treatment of nonallergic chronic rhinitis was well tolerated [59]. A phase I clinical study of a glutathione nasal spray concluded the therapy has a good safety/tolerability profile and is associated with an improvement in clinical symptoms of PD [132]. This study utilized magnetic resonance spectroscopy (MRS) to measure real-time glutathione concentrations in the brain, and demonstrated that intranasal administration of glutathione elevates the brain glutathione level.

### MitoQ & SKQ1

Mitoquinone (MitoQ) is a synthetic powerful mitochondria-targeted antioxidant compound. There is a strong body of evidence indicating the critical role that oxidative stress plays in secondary brain damage mechanisms, such as mitochondrial dysfunction, apoptosis, and inflammatory response following the TBI. MitoQ can defend against this oxidative stress associated with secondary TBI pathogenesis. MitoQ is composed of a lipophilic triphenylphosphonium (TPP) cation to facilitate its penetration into the mitochondria and is soluble in solvent dimethyl sulfoxide (DMSO). A compound with a similar mitochondrion targeting TPP moiety, SKQ1, has been tested intranasally with a result showing a high level of penetration into the brain tissue [61, 133]. This bodes well for intranasal testing of MitoQ as a TBI therapeutic. MitoQ has shown positive outcomes in the animal models of PD, Alzheimer's Disease (AD) and TBI [134–137]. Preliminary safety studies of MitoQ in humans indicated that MitoQ is safe and well-tolerated [138].

### Curcumin

Curcumin is an active component in the spice of turmeric and in Curcuma Xanthorrhiza oil [139]. Its pharmacological properties include anti-inflammatory, anti-tumor, and antioxidant effects. Preclinical studies have identified that therapeutically achievable curcumin concentration protects mitochondria from oxidative damage and attenuates neuronal apoptosis following TBI. Curcumin is a lipophilic compound that displays poor GI absorption and is rapidly metabolized when administered orally [140]. However, intranasal delivery of curcumin has shown to enhance its brain uptake efficiency in a rodent model of oxidative damage in cortical neurons[141]. Curcumin treatment also markedly prevented cellular glutathione depletion and mitigated intracellular ROS generation [141]. Thus, the intranasal route for curcumin should be further explored for TBI. Studies have supported the preventive effect of curcumin in inhibiting the acute effects of neuroinflammation and cognitive decline in AD [142, 143]. Curcumin and its products are safe when taken orally or applied to the skin in the recommended amounts, thus suitable for daily dietary use as established by the Joint Nations and World Health Organization Expert Committee on Food Additives (JECFA) [144].

### Resveratrol

Resveratrol is another potent antioxidant derived from plants. It is linked to mitochondrial biogenesis through the Sirtuin 1 (SIRT1) metabolic regulatory pathway. The SIRT1 promotes the deactivation and activation of coactivator one alpha, the primary regulator of mitochondrial biogenesis [72]. Preclinical studies have established the protective role of resveratrol in TBI, brain ischemia, PD, and AD [145–147]. Clinical trials have shown that Resveratrol supplementation is safe and well tolerated at different doses, and it modulates neuroinflammation, induces adaptive immunity and attenuates the cognition decline in AD [148–150]. Resveratrol is insoluble in water; however, its esterified form has higher lipophilicity and enhanced solubility. Resveratrol ester is a lipid-soluble neutral compound with high absorption but low bioavailability when taken orally [71]. A dramatic increase in resveratrol levels in the CSF was attained by coating it with chitosan when delivered to the brain via nasal administration [151] demonstrating the utility of BBB penetration enhancers for intranasal drug delivery. Interestingly, this marked increase in CSF bioavailability was achieved without any distribution in the systemic circulation, demonstrating a direct nose-to-brain delivery [151]. Typical BBB penetration-enhancing agents are solvents, co-solvents, ionic and some non-ionic surfactants, selected fatty acids, including oleic acid and certain lipids, and cyclodextrin [152]. Intranasal administration of resveratrol nanoparticle formulation has been shown to reduce retinal ganglion cell loss in a multiple sclerosis model of mouse [153]. Major dietary sources of resveratrol include grapes, wine, berries, cocoa*,* peanuts, and soy.

### Apelin-13

Apelin-13 is a 13 amino acid oligopeptide, a novel compound for targeting mitochondria and downstream secondary injury pathology effects following TBI. It prevents mitochondrial depolarization and apoptotic events [114]. Animal studies have suggested that Apelin-13 attenuates secondary injury after TBI, and exerts a neuroprotective effect by suppressing autophagy, preventing BBB disruption, and alleviating brain edema [154]. Apelin-13 is a basic compound that dissolves well in saline. The intranasal delivery of Apelin-13 provides a noninvasive method for directly administering the peptide therapy to the brain and bypassing the BBB [155]. Apelin-13 remarkably decreased cell death and improved long-term functional recovery in a focal ischemic stroke model of mouse [113]. The intranasal delivery of Apelin-13 may help to address issues related to this peptide’s short plasma half-life, poor bioavailability along with the slow absorption, degradation, and avoiding the drug’s first-pass metabolism in the liver [155].

### Quercetin

The antioxidant quercetin is one of the most abundant polyphenolic flavonoids and displays beneficial biological effects in many diseases. It acts via multiple mechanisms of action, such as modulation of mitochondrial biogenesis, mitochondrial membrane potential, oxidative respiration, and ATP anabolism. However, quercetin’s poor solubility as well as limited oral absorption results in low serum and tissue levels [156, 157]. Quercetin is found in many plants and foods, such as red wine, onions, coffee, leaves, green tea, apples, and berries. Studies have suggested that quercetin exerts neuroprotective effects in brain ischemia and in PD [158, 159]. A recent study evaluating nasal powder derivatives of quercetin-β-cyclodextrin combined with mannitol microparticles for intranasal delivery has reported superior CNS penetration and bioavailability [160, 161]. Significant compound levels were achieved at both brain targeting sites and the bloodstream compared to those after oral delivery, which were negligible. Preparing quercetin’s nano-emulsions and administering them via a noninvasive intranasal route offers the possibility of achieving therapeutic concentrations with potentially robust beneficial effects in the CNS.

### DL-3-n-butylphthalide (NBP)

NBP is a lipid-soluble, alkaline compound that has a long-lasting pharmacologic impact, with a half-life of 11.84 h [116, 162]. NBP is metabolized to various products with different physiological functions. NBP prevents oxidative damage and preserves mitochondrial function. Its broad pharmacologic effects also include inhibiting nerve cell apoptosis, anti-inflammatory response, and anti-thrombotic impact [116]. Due to its potent anti-thrombotic and neuroprotective effects, NBP was approved by the FDA in China to treat ischemic stroke [163]. The complex molecular mechanisms associated with NBP metabolites make it a hot compound for research. Recently, daily intranasal NBP treatment provided protective and neurogenic/angiogenic effects in the post-stroke brain accompanied by functional improvements after a focal ischemic stroke in mice [164]. Testing the effects of NBP administered intranasally in TBI would reveal the prospects for its future use in medicine.

We have also included two potential compounds, i.e., glyceryl triacetate (GTA) and triheptanoin, which so far have not been tested intranasally for any CNS indication. However, they offer excellent therapeutic potential for TBI. There are numerous considerations for selecting these two compounds in the list, including factors such as pH, stability, osmolality, and lipophilicity. Most research studies have shown that intranasally administered lipophilic compounds improve CNS bioavailability and reduce the time for the onset of therapeutic response. The rapid absorption of these lipophilic compounds via the nasal mucosa can be utilized to test the therapeutic potential of GTA and Triheptanoin’s mitochondrial oxidative phosphorylation-enhancing properties in the energy deprivation-related pathogenesis of TBI and CNS diseases.

### Glyceryl triacetate (GTA)

GTA is an FDA-cleared food additive that supplies acetate, a widely active precursor that is converted into acetyl-CoA and is central to mitochondrial energy supply, fatty acid synthesis, and lipid metabolism [165]. GTA is a lipophilic neural compound that gets rapidly absorbed following ingestion and freely crosses the plasma membrane. Drug penetration through the BBB is also favored by it’s lipophilicity. Intranasal GTA can be potentially used to deliver metabolizable acetate to supply fuel to an energy-deprived injured brain. However, GTA was administered at a higher dose during past study [166]. Therefore, it would be challenging to deliver an effective therapeutic concentration through the nostrils as the intranasal dosing volume may be a rate-limiting factor compared to traditional routes.

### Triheptanoin

Like GTA, triheptanoin, the triglyceride of heptanoate, is a promising therapeutic alternative biofuel to improve oxidative phosphorylation and aid ATP generation in TBI. Heptanoate can be metabolized to propionyl-CoA, producing succinyl-CoA after carboxylation and thereby re-filling a key substrate of the tricarboxylic acid (TCA) cycle [167]. A preclinical study suggested that triheptanoin slows motor neuron loss and the onset of motor symptoms in Amyotrophic lateral sclerosis (ALS) mice by improving TCA cycling [167]. Likewise, clinical research identified triheptanoin as a promising therapy for neurodegenerative disorders involving energy deficit pathophysiology [168]. The improvement of a child's neurological status with pyruvate carboxylase deficiency during IV treatment with triheptanoin suggests that C5-ketone bodies (Triheptanoin metabolite) are taken up and used by the brain [169]. The intranasal route of administration can achieve peak efficacious CSF concentrations of triheptanoin and its metabolites to provide energy supplementation in TBI. At the clinical level, this lipophilic compound with basic nature (pH) has been evaluated intranasally in AD [170, 171].

Collectively, the intranasal route of administration has surfaced as a valuable alternative for the delivery of neurotherapeutics with problems of CNS bioavailability. Multiple compounds compiled here can be considered the rational for developing mitochondria-protective nasal formulations for brain injury treatment. The greatest number of studies in this review explored antioxidants, especially plant-derived ones. Damaged brain tissue has lower oxidative metabolic and bioenergetic activities, high production of mitochondrial reactive oxygen metabolites, relatively low levels of antioxidants, and non-replicating nature of neuronal cells. This underpins the importance of targeting mitochondrial antioxidant systems to counteract oxidative stress and brain damage. Several preclinical studies reported that antioxidants diminish oxidative stress and improve brain injury outcomes.

Compounds like Resveratrol, Curcumin, Quercetin, Gallotannin, Ginsenosides, Huperzine A, and Geraniol are naturally occurring phytochemicals antioxidants found in fruits and vegetables. Additionally, NMN can be found in avocados and broccoli, and NAC in onion [172, 173]. A list of these phytochemicals strongly suggests using antioxidants as a possible instrument to prevent oxidative stress on neurological targets. Indeed, phytochemicals have gradually become a hotspot in nutrition research due to a plethora of health benefits and their antioxidative properties. More importantly, phytochemicals are proposed as one of the most promising mitochondria-targeting medicine to preserve the activity and structure of mitochondria and neurons [174]. Phytochemicals affect mitochondrial function and structure by modulating the mitochondrial biosynthesis (mitobiogenesis), dynamics (fission, fusion), transport, and autophagic cleavage of damaged mitochondria (mitophagy) [174]. However, despite this popularity, only limited data regarding the safety of most individual phytochemicals treating CNS diseases are available. Many in vitro studies and data have been collated, but the in vivo efficacy and safety experiments still need to be explored. Toxicological screening is essential for pursuing natural bioactive compounds to be used in drug discovery. Therefore, the development of safer antioxidants from natural sources is desired. In this regard, identifying the best phytochemical extraction methods are of crucial value. Since the product will contain traces of extraction solvent, the solvent should be non-toxic. In fact, the choice of extraction methods has the greatest impact on the bio-composition of both active compounds and matrix components obtained from plant sources. Each phytochemical ingredient listed here requires verifiable scientific evidence and significant scientific agreement. The government should set regulations for phytochemical consumption, and the safety and health claims should be monitored appropriately and researched. The authentic data on the effectiveness of phytochemicals against CNS disease can only be drawn from carefully controlled human studies. If these challenges are addressed, there is tremendous future scope for the intranasal application of phytochemicals, and it holds exciting opportunities.

Molecular weight, lipophilicity, and degree of dissociation are some of the primary properties of phytochemicals that dictate to what rate and extent these compounds will transport from the nasal mucosa to the brain. Unfortunately, most phytochemicals are non-lipids, have limited bioavailability due to their poor solubility and stability characteristics [175]. Hence, formulating studies should be carried out to enhance absorption using innovative formulations such as chitosan, nanoemulsions, polymeric nanoparticles, nanocrystals, and exosomes, which can be administered intranasally. Often, the formulations proposed involve the addition of a mucoadhesive polymer to overcome the problem of nasal clearance [176–178]. As shown by the literature, chitosan, a cellulose-based biopolymer plays a significant role as a penetration enhancer and for the mucoadhesion properties [179]. Chitosan nanoemulsions significantly enhanced the quantity of antioxidant drugs found in the brains of the rats following the nasal administration (5- and 4.5-fold higher than with free medicine and nanoemulsion without chitosan, respectively) [180]. Additionally, histopathological examinations suggested that these nanoemulsions were safe for the nasal mucosa and could preserve the drug's antioxidant capability [180]. Combined results of biochemical and histopathological evaluation verified the superiority of surface modification of phytochemical with chitosan coating could be of great value in management of TBI. We have further discussed other surface engineering approaches in the next section.

## Discussion

Drug delivery across the physiological barriers of the brain is one of the challenging issues in the development of treatment of TBI and CNS disorders. This review elaborates on the significance of electing the appropriate drug delivery route and predicting drug delivery to the CNS. Our main purpose was to lay a foundation based on scientific rationale on which informed decisions could be made when designing and testing mitochondria targeting drug candidates, and development and testing of intranasal drug delivery technology and/or devices in the future preclinical and clinical TBI research. Distinctions between the bioavailability of various drugs based on their route of administration, pharmacological and physiological properties may explain why neuroprotective compounds in TBI studies have not been successful so far at the clinical level. The advancement of mitochondria targeted TBI drugs is currently hindered by the BBB’s selective permeability, which limits the distribution of systemically administered therapeutics to the CNS.

Despite the rapid advancement in the medical field, the neuroprotective medicine evaluation and testing is still in its relative infancy, with numerous challenges and hurdles yet to be overcome. Among the neuroprotective medicine cohort, mitochondria-targeted therapy appears to be a promising treatment approach for TBI and many forms of neurodegenerative diseases. Although neuroprotective medicine has been efficacious in a preclinical setting, patient response rates at the clinical level vary, and only a small subset of the patients within a large cohort respond favorably to these treatments leading to a lack of statistically significant clinical outcomes [181]. This issue is particularly concerning and has become a challenge for researchers aiming to improve the effectiveness of mitochondria targeted TBI therapeutics and patient response rates. Therefore, there is a growing realization that the standard drug delivery method used to administer mitochondria-targeted therapies to the CNS might not be efficient. It is critical to quest for alternative CNS delivery routes to achieve effective drug concentrations in the brain.

The intranasal delivery is a promising drug administration method for treating TBI and CNS diseases. The most considerable promise appears to lie in intranasal delivery of phytochemicals antioxidant compounds such as NMN, resveratrol and mitochondria targeted compound MitoQ. However, other therapeutics, including lipophilic precursors of the mitochondrial Krebs cycle (i.e., TCA cycle), such as GTA and triheptanoin, also have significant therapeutic potential. The intranasal route could be a solution to poor oral absorption of neuroprotective compounds like quercetin, gallotannin and tetrandrine. Compounds with a short half-life, like insulin, apelin-13, ginsenoside Rg3 and cyclin D1 may achieve better bioavailability and expedited onset of action following intranasal administration. Earlier, we indicated higher dosing volume of GTA, and resveratrol could be a limiting factor for intranasal administration since the intranasal dosing volume and absorption surface area are limited. However, it is essential to note that the intranasal route avoids pre-absorption metabolism, first-pass effect, and dilution caused by distribution along with protein binding, indicating that the required intranasal volume may easily be as low as 0.01–1% of oral dosage [182]. There are mounting scientific backings that the delivery of drugs via the intranasal route results in higher CNS concentrations of drugs that cannot cross the BBB and fewer side effects [182]. For neuroprotective mitochondria-targeted drugs with several systemic side effects, such as pitavastatin and pentoxifylline, intranasal drug administration could be a promising option to target the CNS using a lower dose that would minimize systemic exposure, thus decreasing the unwanted adverse systemic effects.

**Fig. 3:**
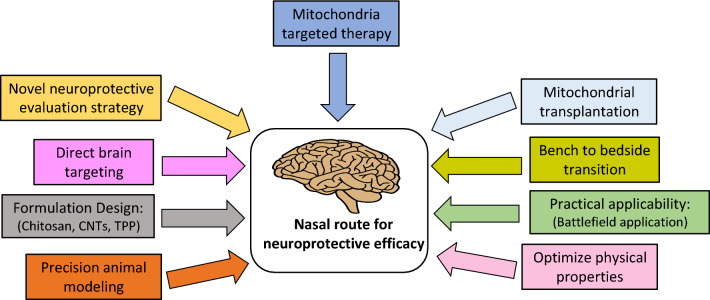
Combination of the ideal parameters to advance nose-to-brain delivery of neuroprotective therapy in TBI

Even with well-established mechanism of action, therapeutic failures of neuroprotective drugs may occur due to lesser absorption in neuronal and other brain cells, slower drug action and conversion of drug molecules into non-interacting metabolites. Due to a time lag in the conventional route of administration, there is a possibility that an active compound may become a slow-acting molecule that may be destroyed once it gets inside the brain tissue or enzyme catalytic activity rendering it useless [183]. Therefore, active penetration, rapid availability, possible structure and activity preservation, and neuroprotective action of a drug in the target area are highly desirable traits for treating TBI and various CNS disorders (Fig. 3). Overall, the future of intranasal delivery for TBI looks promising, as it represents an efficient way for neuroprotective drugs to be delivered quickly, noninvasively, and directly to the brain cells or at the injured site. The quick and noninvasive delivery aspect is crucial if immediate therapy is desired and the patient's ability to deal with injections is impaired, such as in military combat casualty settings. In the civilian setting, intranasal drug delivery could facilitate greater patient compliance with clinical protocols because of the ease of use, which would be an important added benefit.

The combat environment exacerbates the typical challenges of treating medical emergencies [184]. It has additional obstacles, including a lack of supplies and equipment to deliver drugs, delayed or prolonged evacuation times and distances, multiple injuries, provider inexperience, and a dangerous tactical situation [184]. The intranasal delivery can be utilized to alleviate drug delivery challenges on the battlefield to provide prehospital TBI treatment. Thus, intranasal administration offers a noninvasive alternative route of medication delivery when the parental route is unavailable or will result in an unacceptable delay in medication efficacy. Moreover, intranasal administration doesn’t require sterile conditions and hence it can be self-administered in non-sterile battlefield settings. The application of intranasal therapy for combat patients is not limited to TBI and could be used for other acute care management. In keeping with this, the US Army has funded an intranasal clinical trial for ketamine, for pain management in the combat setting, which has shown promising results [185]. Intranasal therapies may be commercially prepared with a built-in atomizer and carried by warfighters.

Notably, targeting mitochondrial pathologies are often mired by efficacy-limiting unintended off-target effects [22]. In addition to the application of the intranasal delivery method, the enrichment of therapeutics at the sub-mitochondrial site of action can reduce deleterious effects and increase therapeutic potency. Mitochondrial drug localization can be accomplished using several approaches, such as the mitochondrial membrane potential, affinity of a carrier to mitochondria-specific components, and nanoparticle-based approaches [22]. MitoQ, an endogenous antioxidant ubiquinone derivative discussed earlier, used mitochondrial membrane potential to target mitochondria. The matrix-negative voltage difference promotes the selective accumulation of cationic compounds such as TPP in mitochondria. TPP conjugates of phytochemical antioxidants such as quercetin, resveratrol, curcumin and NAC were also reported to improve mitochondrial activity [186–188]. Exploring additional methods to increase mitochondrial penetration of intranasally delivered therapeutic agents will pave the way for the next breakthroughs in neurotherapeutics development.

The intranasal delivery signifies a new frontier in CNS disease treatment that has shown promise since its initial conceptualization. However, there is also contrary evidence that the intranasal route is relatively inconvenient to patients compared to oral delivery since nasal irritation is possible [189]. Therefore, each neuroprotective compound must be examined for its safety effects on the nasal mucosa when drug is given intranasally. Besides the information used for appraisal in this review for the selection of intranasal compounds, several other essential characteristics should be considered. These properties include: the intranasal compound should have no unpleasant odor, should not be irritating to nasal mucosa or influence the sense of smell, and should be potent enough so that post-intranasal administration bioavailability of the drug can reach therapeutic efficacy. Additionally, the optimal volume of the intranasal administration is 0.5 to 1 ml per nostril in humans; therefore, the compound formulation must remain within the standard volume range [190, 191]. Furthermore, compounds that are metabolized by enzymes such as peptidase in the nasal cavity must be sheltered from degradation. Studies have also suggested that physical and pathological conditions such as allergies, polyps and the common cold may affect nasal absorption [192].

Understanding the in-depth pharmacology of each neuroprotective compound is essential for the intranasal experimental planning, but it is beyond the scope of this paper to review the in-depth pharmacokinetics and pharmacodynamics (PK-PD) properties of individual compounds. Before conducting animal studies, the research team members should receive proper intranasal route drug delivery training and attain competency. Experimental issues such as the selection of animals, volume of administration, use of anesthesia, and pH of the substance must be refined for the intranasal delivery. The intranasal administration in rats typically requires anesthesia, although devices are being developed to circumvent this limitation. However, intranasal drug delivery to mice can easily be done using a pipette or readily available atomization devices without any anesthesia, making mice the ideal first-line test subjects. Importantly, adequate training and thoughtfulness to details is important to mitigate inadvertent adverse effects on animal health and confounded experimental outcomes when testing the intranasal method of administration [193].

Also, there are factors such as differences in the nasal anatomy and physiology of animal species and humans, making it difficult to obtain a direct correlation between them when translating preclinical findings to clinical research. Therefore, it is imperative to study the anatomy of the animal's nasal cavity before electing appropriate animal models for the intranasal studies. The key to overcoming these challenges and advancing the field of intranasal drug delivery is to develop informative methodologies to better understand the nose-to-brain delivery pathway. Elucidating the drug pathways after intranasal administration is central to develop relevant drug delivery systems for intranasal approaches. A critical tool at our disposal is in vivo imaging to track a drug's route. Imaging has excellent potential to facilitate the translation of promising intranasal therapies from animals to humans, and improved imaging techniques continue to emerge [194].

The feasibility of intranasal drug delivery is limited in the context of a patient with a skull fracture impacting the nasal cavity or cribriform plate, a history of coagulopathy disorder or friable (crumbly) nasal mucosa. In addition, congestion, bleeding, or obstruction in the nose following TBI may prevent intranasal administration. Although dozens of studies have tested intranasal delivery of mitochondria-targeting compounds preclinically, it is surprising that nanotechnology has not been extensively explored for this promising route. Recently, multiple systems have been successfully formulated using nanomaterials for intranasal delivery. A nanotechnology-based delivery system like chitosan, carbon nanotubes (CNTs), and polylactic-co-glycolic acid (PLGA) have been studied in vitro and in vivo for the delivery of several therapeutic agents and have shown promising concentrations in the brain after nasal administration [195]. Nanotechnology offers great potential to enhance drug penetration through the nasal barrier at a minimal volume, and without altering physiochemical properties for better absorption.

CNTs are the strongest candidates in the province of nanobiotechnology and nanomedicine, promising to treat various CNS diseases. A CNT is a tube made of carbon atoms organized in a series of condensed benzene rings with a diameter in the nanometer range that can penetrate BBB [196]. CNTs can be either single-walled or multi-walled, with open ends, or maybe closed with fullerene caps [196]. Their unique surface area, hollow drug-loadable central cavities, strength, and resilience have led to much excitement in the pharmacy field. Utilizing an easily modifiable surface, many therapeutic molecules have been incorporated into the functionalized CNTs for delivery to the site of interest [197]. Conjugating mitochondria-targeted compounds with CNT is one of the surface engineering approaches that can improve its intranasal absorption and open the way for the effective brain-targeting delivery. Transportation of encapsulated drugs across the nasal membrane, lengthening the retention period, and higher stability can complement the overall intranasal absorption. Recently, multi-walled carbon nanotubes have been found to exert neuroprotective effects by modulating vital neurotrophic factors when delivered via the intranasal route [198]. Despite CNT's promising outlook, they have some limitations, such as protein corona formation around them and cytotoxic effects. Studies have reported that systemic administration of CNTs are often associated with severe CNS toxicity; the interaction of CNT with brain cells leads to the release of mediators from microglia and astrocytes that may result in apoptosis, inflammation and oxidative stress in the brain [199, 200].

Therefore, the functionalization of nanocarriers on their interaction with brain tissue is deemed critical for developing nanotube-based intranasal delivery for CNS applications. Owing to the small number of nanocarbons required through intranasal delivery, and the even smaller amount of material released from implanted complexes; intranasal application of nanocarbon may mitigate its practical application challenges due to dose-related CNS toxicity. However, to accomplish competent drug delivery, it is imperative to recognize the interactions of CNT and the nasal biological environment, drug release, multiple drug administration, and stability of therapeutic compounds. The biosafety of each therapeutic modality must be demonstrated in logical and well-conducted experiments. The promising combination of nanocarriers and intranasal delivery needs to elucidate better clinical, pharmacokinetics, and safety profiles. Although there are some hurdles in its clinical application, the success of the CNTs may result in the development of a new and highly relevant drug delivery procedure benefiting several patients in the near future.

Additionally, the nose-to-brain pathway may enable the rapid delivery of mitochondria to the CNS within minutes. Autologous mitochondrial replacement therapy with parental administration and direct injection has been employed to treat mitochondrial diseases in clinical trials [201]. Mitochondria are nano-sized cell organelles measuring approximately 200–1000 nm in size [202]; thus, they can penetrate the nasal mucosa. A study has shown that mitochondria can enter brain meninges and parenchyma upon nasal delivery and undergo rapid cellular internalization [203]. Recent evidence has indicated that the physiological properties of healthy mitochondria provide the possibility of replacing damaged mitochondria [204], suggesting that the replacement of damaged mitochondria with healthy mitochondria may protect cells against further injury following TBI [205]. The intranasal administration of mitochondria can be explored as an effective transplantation strategy of fully functional mitochondria directly into defective neurons, reversing TBI pathogenesis and restoring brain energy supplements.

Several drugs tested for CNS disorders and TBI were discarded despite the well-established mitochondrial-enhancing mechanisms because their efficiency is marred due to the presence of the BBB. The intranasal delivery route may well revive further research on these mitochondria targeting neuroprotective drugs. The intranasal pathway offers a unique opportunity to repurpose old drugs for new uses and to improve the efficacy of currently approved medications indicated for other administration routes [206]. To facilitate and expedite TBI therapeutic development, intranasal delivery of neuroprotective experimental compounds, including the list provided here can play a critical role.

The promising results of numerous intranasal deliveries reported in this review do not allow these findings to be equalized to human use, and selecting promising compounds. They must be established by additional research and further experiments. Therefore, significant amount of future research needed to translate these experimental results from bench to bedside. In this regard, utilizing more representative larger animal models (e.g., non-human primates, swine, ferrets) in parallel with rodents for intranasal delivery research could improve the predictive value of preclinical studies. Non-human primates would be more accurate animal model due to their greater anatomical and physiological resemblance to humans. There were relatively few direct comparative studies on animal models and human counterparts in terms of their nasal anatomy and physiology, and these studies would have been valuable in clarifying the specific similarities and differences between the two species. Although the olfactory pathway to the brain in humans is well established, it remains an area to explore and understand as to what extent it contributes to the CNS availability of compounds administered via the nasal route. The key to furthering the field of intranasal delivery is to develop methodologies to better understand the nose-to-brain drug delivery pathways. Since only a small quantity of drugs are delivered to the brain intranasally, the mechanisms of drug delivery need to be better clarified, and novel methods need to be developed to overcome the obstacles facing nose-to-brain delivery of promising mitochondrial therapeutics. Hopefully, future clinical studies will be conducted on neuroprotective drugs reported in this review by utilizing physicochemical and pharmacokinetic properties of drugs to treat TBI and other CNS diseases. Using the nose-to-brain route to overcome listed pharmacokinetic challenges will allow future studies to better elucidate the neuroprotective efficacy of mitochondria-targeted drugs on CNS.

## Conclusion

The approach aimed at mitochondrial-targeted drug delivery is achievable through the intranasal route. Shortcomings associated with most of the neuroprotective compounds compiled in this review when administered through a conventional route, such as low bioavailability due to BBB, drug degradation in the GI tract, and first-pass metabolism, make them ideal candidates for the intranasal administration. Our study indicated that post-TBI intranasal administration of the mitochondria-targeted neuroprotective compound appears to be a promising strategy to bypass the BBB. Practically, the intranasal drug administration offers several benefits for patients, as it represents a noninvasive, painless, simple drug delivery system, which is manageable and easily repeatable. By localizing drugs at their desired site of action, systemic toxicity can be reduced, and treatment efficiency can be increased. However, there are still formulation limitations, and toxicological aspects to be optimized. Further study of this clinically relevant route of administration for mitochondria-targeting compounds is warranted in TBI animal models to optimize this route and fully understand dosing, therapeutic window and safety issues related to the route.

## Data Availability

Not applicable to this article as no new data were created or analyzed in this study.

## Abbreviations

TBI: Traumatic brain injury
AD: Alzheimer’s disease
PD: Parkinson’s disease
BBB: Blood–brain barrier
BCB: Blood-CNS barrier
BCSFB: Blood-CSF barrier
TJ: Tight junctions
CNS: Central nervous system
CSF: Cerebral spinal fluid
CDC: Centers for disease control and prevention
GI: Gastrointestinal
ICV: Intracerebroventricular
IV: Intravenous
IP: Intraperitoneal
IM: Intramuscular
HMG CoA: β-Hydroxy β-methylglutaryl coenzyme A
MOA: Mechanism of action
GTA: Glycerol triacetate
FDA: Food and Drug Administration
TCA: Tricarboxylic acid
ETC: Electron transport chain
ROS: Reactive oxygen species
ALS: Amyotrophic lateral sclerosis
ATP: Adenosine triphosphate
NMN: Nicotinamide mononucleotide
NAD: Nicotinamide adenine dinucleotide
NACA: n-acetylcysteine amide
NAC: n-acetylcysteine
DMSO: Dimethyl sulfoxide
SIRT1: Sirtuin 1
PARP: Poly-ADP ribose polymerase
QUR: Quercetin
NBP: DL-3-n-butylphthalide
CNT: Carbon nanotubes
PLGA: Polylactic-co-glycolic acid

## References

[1] Markgraf CG, Clifton GL, Moody MR (2001). Treatment window for hypothermia in brain injury. J Neurosurg.

[2] Baratz-Goldstein R, Toussia-Cohen S, Elpaz A, Rubovitch V, Pick CG (2017). Immediate and delayed hyperbaric oxygen therapy as a neuroprotective treatment for traumatic brain injury in mice. Mol Cell Neurosci.

[3] Thurman DJ, Alverson C, Dunn KA, Guerrero J, Sniezek JE (1999). Traumatic brain injury in the United States: a public health perspective. J Head Trauma Rehabil.

[4] Georges A. Traumatic brain injury. 2017.

[5] Ghajar J (2000). Traumatic brain injury. The Lancet.

[6] Stein DG (2015). Embracing failure: what the phase III progesterone studies can teach about TBI clinical trials. Brain Inj.

[7] Dickey N, Jenkins D, Butler FK. Prehospital Use of Ketamine in Battlefield Analgesia 2012–03. Falls Church, Virginia USA: Defense Health Board, Memorandum. 2012.

[8] Andriessen TM, Jacobs B, Vos PE (2010). Clinical characteristics and pathophysiological mechanisms of focal and diffuse traumatic brain injury. J Cell Mol Med.

[9] Dash PK, Zhao J, Hergenroeder G, Moore AN (2010). Biomarkers for the diagnosis, prognosis, and evaluation of treatment efficacy for traumatic brain injury. Neurotherapeutics.

[10] Marklund N, Bakshi A, Castelbuono DJ, Conte V, McIntosh TK (2006). Evaluation of pharmacological treatment strategies in traumatic brain injury. Curr Pharm Des.

[11] Ma X, Aravind A, Pfister BJ, Chandra N, Haorah J (2019). Animal models of traumatic brain injury and assessment of injury severity. Mol Neurobiol.

[12] Freire MAM, Rocha GS, Bittencourt LO, Falcao D, Lima RR, Cavalcanti J (2023). Cellular and molecular pathophysiology of traumatic brain injury: what have we learned so far?. Biology.

[13] Hubbard WB, Joseph B, Spry M, Vekaria HJ, Saatman KE, Sullivan PG (2019). Acute mitochondrial impairment underlies prolonged cellular dysfunction after repeated mild traumatic brain injuries. J Neurotrauma.

[14] Kilbaugh TJ, Karlsson M, Byro M, Bebee A, Ralston J, Sullivan S (2015). Mitochondrial bioenergetic alterations after focal traumatic brain injury in the immature brain. Exp Neurol.

[15] Pandya JD, Pauly JR, Nukala VN, Sebastian AH, Day KM, Korde AS (2007). Post-injury administration of mitochondrial uncouplers increases tissue sparing and improves behavioral outcome following traumatic brain injury in rodents. J Neurotrauma.

[16] Sullivan PG, Rabchevsky AG, Keller JN, Lovell M, Sodhi A, Hart RP (2004). Intrinsic differences in brain and spinal cord mitochondria: implication for therapeutic interventions. J Comp Neurol.

[17] Pandya JD, Leung LY, Hwang HM, Yang X, Deng-Bryant Y, Shear DA (2021). Time-course evaluation of brain regional mitochondrial bioenergetics in a pre-clinical model of severe penetrating traumatic brain injury. J Neurotrauma.

[18] Zhang HT, Zhang P, Gao Y, Li CL, Wang HJ, Chen LC (2017). Early VEGF inhibition attenuates blood-brain barrier disruption in ischemic rat brains by regulating the expression of MMPs. Mol Med Rep.

[19] Fiskum G, Rosenthal RE, Vereczki V, Martin E, Hoffman GE, Chinopoulos C (2004). Protection against ischemic brain injury by inhibition of mitochondrial oxidative stress. J Bioenerg Biomembr.

[20] Cheng G, Kong R, Lm Z, Jn Z (2012). Mitochondria in traumatic brain injury and mitochondrial-targeted multipotential therapeutic strategies. Br J Pharmacol.

[21] Pandya JD, Leung LY, Flerlage WJ, Gilsdorf JS, Bryant YD, Shear D (2019). Comprehensive profile of acute mitochondrial dysfunction in a preclinical model of severe penetrating TBI. Front Neurol.

[22] Lamade AM, Kenny EM, Anthonymuthu TS, Soysal E, Clark RSB, Kagan VE (2019). Aiming for the target: Mitochondrial drug delivery in traumatic brain injury. Neuropharmacology.

[23] Pardridge WM (2012). Drug transport across the blood-brain barrier. J Cereb Blood Flow Metab.

[24] Pardridge WM (2007). Blood-brain barrier delivery. Drug Discov Today.

[25] Ghose AK, Herbertz T, Hudkins RL, Dorsey BD, Mallamo JP (2012). Knowledge-based, central nervous system (CNS) lead selection and lead optimization for CNS drug discovery. ACS Chem Neurosci.

[26] Pardridge WM (2005). The blood-brain barrier: bottleneck in brain drug development. NeuroRx.

[27] Islam SU, Shehzad A, Ahmed MB, Lee YS (2020). intranasal delivery of nanoformulations: a potential way of treatment for neurological disorders. Molecules.

[28] Almazroo OA, Miah MK, Venkataramanan R (2017). Drug metabolism in the liver. Clin Liver Dis.

[29] Anderson GD, Gidal BE, Hendryx RJ, Awan AB, Temkin NR, Wilensky AJ (1994). Decreased plasma protein binding of valproate in patients with acute head trauma. Br J Clin Pharmacol.

[30] Hanson LR, Frey WH (2007). Strategies for intranasal delivery of therapeutics for the prevention and treatment of neuroAIDS. J Neuroimmune Pharmacol.

[31] Liu XF, Fawcett JR, Thorne RG, DeFor TA, Frey WH (2001). Intranasal administration of insulin-like growth factor-I bypasses the blood-brain barrier and protects against focal cerebral ischemic damage. J Neurol Sci.

[32] Thorne RG, Pronk GJ, Padmanabhan V, Frey WH (2004). Delivery of insulin-like growth factor-I to the rat brain and spinal cord along olfactory and trigeminal pathways following intranasal administration. Neuroscience.

[33] Ross TM, Martinez PM, Renner JC, Thorne RG, Hanson LR, Frey WH (2004). Intranasal administration of interferon beta bypasses the blood-brain barrier to target the central nervous system and cervical lymph nodes: a non-invasive treatment strategy for multiple sclerosis. J Neuroimmunol.

[34] Danielyan L, Schäfer R, von Ameln-Mayerhofer A, Buadze M, Geisler J, Klopfer T (2009). Intranasal delivery of cells to the brain. Eur J Cell Biol.

[35] Benedict C, Brede S, Schiöth HB, Lehnert H, Schultes B, Born J (2011). Intranasal insulin enhances postprandial thermogenesis and lowers postprandial serum insulin levels in healthy men. Diabetes.

[36] Freiherr J, Hallschmid M, Frey WH, Brünner YF, Chapman CD, Hölscher C (2013). Intranasal insulin as a treatment for Alzheimer’s disease: a review of basic research and clinical evidence. CNS Drugs.

[37] Hanson LR, Fine JM, Svitak AL, Faltesek KA (2013). Intranasal administration of CNS therapeutics to awake mice. J Vis Exp.

[38] Chapman CD, Frey WH, Craft S, Danielyan L, Hallschmid M, Schiöth HB (2013). Intranasal treatment of central nervous system dysfunction in humans. Pharm Res.

[39] MacDonald E, Dadds MR, Brennan JL, Williams K, Levy F, Cauchi AJ (2011). A review of safety, side-effects and subjective reactions to intranasal oxytocin in human research. Psychoneuroendocrinology.

[40] Nathan RA (2011). Intranasal steroids in the treatment of allergy-induced rhinorrhea. Clin Rev Allergy Immunol.

[41] Shemesh E, Rudich A, Harman-Boehm I, Cukierman-Yaffe T (2012). Effect of intranasal insulin on cognitive function: a systematic review. J Clin Endocrinol Metab.

[42] Wolfe TR, Macfarlane TC (2006). Intranasal midazolam therapy for pediatric status epilepticus. Am J Emerg Med.

[43] Reese TS, Feder N, Brightman MW (1971). Electron microscopic study of the blood-brain and blood-cerebrospinal fluid barriers with microperoxidase. J Neuropathol Exp Neurol.

[44] Nau R, Sörgel F, Eiffert H (2010). Penetration of drugs through the blood-cerebrospinal fluid/blood-brain barrier for treatment of central nervous system infections. Clin Microbiol Rev.

[45] Price L, Wilson C, Grant G (2016). Blood–brain barrier pathophysiology following traumatic brain injury. Transl Res Trauma Brain Injury.

[46] Beaumont A, Fatouros P, Gennarelli T, Corwin F, Marmarou A (2006). Bolus tracer delivery measured by MRI confirms edema without blood-brain barrier permeability in diffuse traumatic brain injury. Acta Neurochir Suppl.

[47] Barzó P, Marmarou A, Fatouros P, Corwin F, Dunbar J (1996). Magnetic resonance imaging-monitored acute blood-brain barrier changes in experimental traumatic brain injury. J Neurosurg.

[48] Lopez NE, Krzyzaniak MJ, Blow C, Putnam J, Ortiz-Pomales Y, Hageny AM (2012). Ghrelin prevents disruption of the blood-brain barrier after traumatic brain injury. J Neurotrauma.

[49] Lv Q, Fan X, Xu G, Liu Q, Tian L, Cai X (2013). Intranasal delivery of nerve growth factor attenuates aquaporins-4-induced edema following traumatic brain injury in rats. Brain Res.

[50] Chodobski A, Zink BJ, Szmydynger-Chodobska J (2011). Blood-brain barrier pathophysiology in traumatic brain injury. Transl Stroke Res.

[51] Menge T, Zhao Y, Zhao J, Wataha K, Gerber M, Zhang J (2012). Mesenchymal stem cells regulate blood-brain barrier integrity through TIMP3 release after traumatic brain injury. Sci Transl Med..

[52] Chen J, Hu J, Liu H, Xiong Y, Zou Y, Huang W (2018). FGF21 protects the blood-brain barrier by upregulating PPARγ via FGFR1/β-klotho after traumatic brain injury. J Neurotrauma.

[53] Doll DN, Hu H, Sun J, Lewis SE, Simpkins JW, Ren X (2015). Mitochondrial crisis in cerebrovascular endothelial cells opens the blood-brain barrier. Stroke.

[54] Pathan SA, Iqbal Z, Zaidi S, Talegaonkar S, Vohra D, Jain GK (2009). CNS drug delivery systems: novel approaches. Recent Pat Drug Deliv Formul.

[55] Sarkar MA (1992). Drug metabolism in the nasal mucosa. Pharm Res.

[56] Manallack DT (2007). The pK(a) distribution of drugs: application to drug discovery. Perspect Med Chem.

[57] Farina DJ (2010). Regulatory aspects of nasal and pulmonary spray drug products.

[58] Ying W, Wei G, Wang D, Wang Q, Tang X, Shi J (2007). Intranasal administration with NAD+ profoundly decreases brain injury in a rat model of transient focal ischemia. Front Biosci.

[59] Maffezzoni E, Notargiacomo M, Agostini S, Gelardi M (2020). Efficacy of a nasal spray containing N-acetylcysteine in hypertonic solution in the treatment of nonallergic chronic rhinitis with goblet cell metaplasia. J Biol Regul Homeost Agents.

[60] Kawoos U, McCarron RM, Chavko M (2017). Protective effect of N-acetylcysteine amide on blast-induced increase in intracranial pressure in rats. Front Neurol.

[61] Silachev DN, Plotnikov EY, Zorova LD, Pevzner IB, Sumbatyan NV, Korshunova GA (2015). Neuroprotective effects of mitochondria-targeted plastoquinone and thymoquinone in a rat model of brain ischemia/reperfusion injury. Molecules.

[62] Li Y, Fawcett JP, Zhang H, Tucker IG (2007). Transport and metabolism of MitoQ10, a mitochondria-targeted antioxidant, in Caco-2 cell monolayers. J Pharm Pharmacol.

[63] Guo C, Li M, Qi X, Lin G, Cui F, Li F (2016). Intranasal delivery of nanomicelle curcumin promotes corneal epithelial wound healing in streptozotocin-induced diabetic mice. Sci Rep.

[64] Chauhan PS, Singh DK, Dash D, Singh R (2018). Intranasal curcumin regulates chronic asthma in mice by modulating NF-ĸB activation and MAPK signaling. Phytomedicine.

[65] Chen X, Zhi F, Jia X, Zhang X, Ambardekar R, Meng Z (2013). Enhanced brain targeting of curcumin by intranasal administration of a thermosensitive poloxamer hydrogel. J Pharm Pharmacol.

[66] Kumari A, Dash D, Singh R (2015). Lipopolysaccharide (LPS) exposure differently affects allergic asthma exacerbations and its amelioration by intranasal curcumin in mice. Cytokine.

[67] Kumari A, Tyagi N, Dash D, Singh R (2015). Intranasal curcumin ameliorates lipopolysaccharide-induced acute lung injury in mice. Inflammation.

[68] Zhuang X, Xiang X, Grizzle W, Sun D, Zhang S, Axtell RC (2011). Treatment of brain inflammatory diseases by delivering exosome encapsulated anti-inflammatory drugs from the nasal region to the brain. Mol Ther.

[69] Monteillier A, Voisin A, Furrer P, Allémann E, Cuendet M (2018). Intranasal administration of resveratrol successfully prevents lung cancer in A/J mice. Sci Rep.

[70] Shamsher E, Sulaimankutty R, Dine K, Luong V, Davis B, Willett K, et al. Intranasal delivery of resveratrol nanoparticles reduces retinal ganglion cell loss in a model of multiple sclerosis. 2020;61(7):2476

[71] Bastianetto S, Ménard C, Quirion R (2015). Neuroprotective action of resveratrol. Biochim Biophys Acta Mol Basis Dis.

[72] Ungvari Z, Sonntag WE, de Cabo R, Baur JA, Csiszar A (2011). Mitochondrial protection by resveratrol. Exerc Sport Sci Rev.

[73] Rompicherla SKL, Arumugam K, Bojja SL, Kumar N, Rao CM (2021). Pharmacokinetic and pharmacodynamic evaluation of nasal liposome and nanoparticle based rivastigmine formulations in acute and chronic models of Alzheimer’s disease. Naunyn Schmiedebergs Arch Pharmacol.

[74] Bhanderi M, Shah J, Gorain B, Nair AB, Jacob S, Asdaq SMB (2021). Optimized rivastigmine nanoparticles coated with eudragit for intranasal application to brain delivery: evaluation and nasal ciliotoxicity studies. Materials.

[75] EXELON. https://www.accessdata.fda.gov/drugsatfda_docs/label/2000/21025lbl.pdf.

[76] Kandiah N, Pai MC, Senanarong V, Looi I, Ampil E, Park KW (2017). Rivastigmine: the advantages of dual inhibition of acetylcholinesterase and butyrylcholinesterase and its role in subcortical vascular dementia and Parkinson’s disease dementia. Clin Interv Aging.

[77] Saha P, Gupta R, Sen T, Sen N (2018). Activation of cyclin D1 affects mitochondrial mass following traumatic brain injury. Neurobiol Dis.

[78] Kanie T, Onoyama I, Matsumoto A, Yamada M, Nakatsumi H, Tateishi Y (2012). Genetic reevaluation of the role of F-box proteins in cyclin D1 degradation. Mol Cell Biol.

[79] Iqubal A, Sharma S, Sharma K, Bhavsar A, Hussain I, Iqubal MK (2018). Intranasally administered pitavastatin ameliorates pentylenetetrazol-induced neuroinflammation, oxidative stress and cognitive dysfunction. Life Sci.

[80] Ashhar MU, Ahmad MZ, Jain V, Agarwal NB, Ahmad FJ, Jain GK (2017). Intranasal pitavastatin attenuates seizures in different experimental models of epilepsy in mice. Epilepsy Behav.

[81] Livalo. https://www.rxlist.com/livalo-side-effects-drug-center.htm.

[82] Kurata T, Miyazaki K, Morimoto N, Kawai H, Ohta Y, Ikeda Y (2013). Atorvastatin and pitavastatin reduce oxidative stress and improve IR/LDL-R signals in Alzheimer’s disease. Neurol Res.

[83] Kajinami K, Takekoshi N, Saito Y (2003). Pitavastatin: efficacy and safety profiles of a novel synthetic HMG-CoA reductase inhibitor. Cardiovasc Drug Rev.

[84] Zhao N, Zhuo X, Lu Y, Dong Y, Ahmed ME, Tucker D (2017). Intranasal delivery of a caspase-1 inhibitor in the treatment of global cerebral ischemia. Mol Neurobiol.

[85] Pirzada RH, Javaid N, Choi S (2020). The roles of the NLRP3 inflammasome in neurodegenerative and metabolic diseases and in relevant advanced therapeutic interventions. Genes.

[86] Yang H, Gu Z-T, Li L, Maegele M, Zhou B-Y, Li F (2017). SIRT1 plays a neuroprotective role in traumatic brain injury in rats via inhibiting the p38 MAPK pathway. Acta Pharmacol Sinica.

[87] Musumeci T, Bonaccorso A, Puglisi G (2019). Epilepsy disease and nose-to-brain delivery of polymeric nanoparticles: an overview. Pharmaceutics.

[88] PENTOXIFYLLINE. https://dailymed.nlm.nih.gov/dailymed/fda/fdaDrugXsl.cfm?setid=7096593b-0054-4d51-83df-0208ccdcc147&type=display.

[89] Kang Y, Yan W, Fang H, Zhang G, Du Y, Wang L (2017). Alleviation of oxidative damage and involvement of Nrf2-ARE pathway in mesodopaminergic system and hippocampus of status epilepticus rats pretreated by intranasal pentoxifylline. Oxid Med Cell Longev.

[90] Pentoxifylline. https://pubchem.ncbi.nlm.nih.gov/compound/Pentoxifylline.

[91] Naidoo V, Mdanda S, Ntshangase S, Naicker T, Kruger HG, Govender T (2019). Brain penetration of ketamine: Intranasal delivery VS parenteral routes of administraion. J Psychiatr Res.

[92] Cromhout A (2003). Ketamine: its use in the emergency department. Emerg Med.

[93] Liang J, Wu S, Xie W, He H (2018). Ketamine ameliorates oxidative stress-induced apoptosis in experimental traumatic brain injury via the Nrf2 pathway. Drug Des Devel Ther.

[94] Zhang L, Pang L, Zhu S, Ma J, Li R, Liu Y (2020). Intranasal tetrandrine temperature-sensitive in situ hydrogels for the treatment of microwave-induced brain injury. Int J Pharm.

[95] Xu M, Sheng L, Zhu X, Zeng S, Chi D, Zhang G-J (2010). Protective effect of tetrandrine on doxorubicin-induced cardiotoxicity in rats. Tumori J.

[96] Salameh TS, Bullock KM, Hujoel IA, Niehoff ML, Wolden-Hanson T, Kim J (2015). Central nervous system delivery of intranasal insulin: mechanisms of uptake and effects on cognition. J Alzheimers Dis.

[97] Lioutas V-A, Alfaro-Martinez F, Bedoya F, Chung C-C, Pimentel DA, Novak V (2015). Intranasal insulin and insulin-like growth factor 1 as neuroprotectants in acute ischemic stroke. Transl Stroke Res.

[98] Ruegsegger GN, Manjunatha S, Summer P, Gopala S, Zabeilski P, Dasari S (2019). Insulin deficiency and intranasal insulin alter brain mitochondrial function: a potential factor for dementia in diabetes. FASEB J.

[99] Hölscher C (2020). Brain insulin resistance: role in neurodegenerative disease and potential for targeting. Expert Opin Investig Drugs.

[100] Montgomery MK, Turner N (2015). Mitochondrial dysfunction and insulin resistance: an update. Endocr Connect.

[101] Ms S, Ms N, Mmi B, Me-S M, Mae-H S (2020). Novel intranasal drug delivery: geraniol charged polymeric mixed micelles for targeting cerebral insult as a result of ischaemia/reperfusion. Pharmaceutics.

[102] Remington JP (2006). The science and practice of pharmacy.

[103] Soliman MS, Sheta MN, Ibrahim MMB, El-Shawwa MM, Abd El-Halim MS (2020). Novel intranasal drug delivery: geraniol charged polymeric mixed micelles for targeting cerebral insult as a result of ischaemia/reperfusion. Pharmaceutics.

[104] Rekha KR, Sivakamasundari RI (2018). Geraniol protects against the protein and oxidative stress induced by rotenone in an in vitro model of Parkinson’s disease. Neurochem Res.

[105] Jiang T, Huang L, Zhang X, Liang XJIJCEM (2019). Nasal administration of muscone promotes cAMP-PKA-CREB signaling in rats with traumatic brain injury. Int J Clin Exp Med.

[106] Ly Z, Yao M, Zr T, Sf L, Yj S, Ye J (2020). Muscone suppresses inflammatory responses and neuronal damage in a rat model of cervical spondylotic myelopathy by regulating Drp1-dependent mitochondrial fission. J Neurochem.

[107] Jiang T, Huang L, Zhang X, Liang X (2019). Nasal administration of muscone promotes cAMP-PKA-CREB signaling in rats with traumatic brain injury. Int J Clin Exp Med.

[108] Boxer AL, Lang AE, Grossman M, Knopman DS, Miller BL, Schneider LS (2014). Davunetide in patients with progressive supranuclear palsy: a randomised, double-blind, placebo-controlled phase 2/3 trial. Lancet Neurol.

[109] Magen I, Ostritsky R, Richter F, Zhu C, Fleming SM, Lemesre V (2014). Intranasal NAP (davunetide) decreases tau hyperphosphorylation and moderately improves behavioral deficits in mice overexpressing α-synuclein. Pharmacol Res Perspect.

[110] Davunetide. https://pubchem.ncbi.nlm.nih.gov/compound/9832404#section=Pharmacology-and-Biochemistry.

[111] Arya A, Meena R, Sethy NK, Das M, Sharma M, Bhargava K (2015). NAP (davunetide) protects primary hippocampus culture by modulating expression profile of antioxidant genes during limiting oxygen conditions. Free Radic Res.

[112] Gold M, Lorenzl S, Stewart AJ, Morimoto BH, Williams DR, Gozes I (2012). Critical appraisal of the role of davunetide in the treatment of progressive supranuclear palsy. Neuropsychiatr Dis Treat.

[113] Chen D, Lee J, Gu X, Wei L, Yu SP (2015). Intranasal delivery of Apelin-13 Is neuroprotective and promotes angiogenesis after ischemic stroke in mice. ASN Neuro.

[114] Zeng X, Yu SP, Taylor T, Ogle M, Wei L (2012). Protective effect of apelin on cultured rat bone marrow mesenchymal stem cells against apoptosis. Stem Cell Res.

[115] Ahmad N, Ahmad R, Naqvi AA, Alam MA, Ashafaq M, Abdur Rub R (2018). Intranasal delivery of quercetin-loaded mucoadhesive nanoemulsion for treatment of cerebral ischaemia. Artif Cells Nanomed Biotechnol.

[116] Chen X-Q, Qiu K, Liu H, He Q, Bai J-H, Lu W (2019). Application and prospects of butylphthalide for the treatment of neurologic diseases. Chin Med J.

[117] Wei ZZ, Chen D, Lee MJH, Zhao Y, Gu X, Yu SP (2021). DL-3-n-butylphthalide increases collateriogenesis and functional recovery after focal ischemic stroke in mice. Aging Dis.

[118] Wei G, Wang D, Lu H, Parmentier S, Wang Q, Panter SS (2007). Intranasal administration of a PARG inhibitor profoundly decreases ischemic brain injury. Front Biosci.

[119] Ducharme N, Banks WA, Morley JE, Robinson SM, Niehoff ML, Mattern C (2010). Brain distribution and behavioral effects of progesterone and pregnenolone after intranasal or intravenous administration. Eur J Pharmacol.

[120] Sitruk-Ware R (2018). Non-clinical studies of progesterone. Climacteric.

[121] Irwin RW, Yao J, Hamilton RT, Cadenas E, Brinton RD, Nilsen J (2008). Progesterone and estrogen regulate oxidative metabolism in brain mitochondria. Endocrinology.

[122] Meng Q, Wang A, Hua H, Jiang Y, Wang Y, Mu H (2018). Intranasal delivery of Huperzine A to the brain using lactoferrin-conjugated N-trimethylated chitosan surface-modified PLGA nanoparticles for treatment of Alzheimer’s disease. Int J Nanomedicine.

[123] Zhao Y, Yue P, Tao T, Chen QH (2007). Drug brain distribution following intranasal administration of Huperzine A in situ gel in rats. Acta Pharmacol Sin.

[124] Li Y, Zhang R, Li C, Jiang X (2007). Pharmacokinetics of huperzine A following oral administration to human volunteers. Eur J Drug Metab Pharmacokinet.

[125] Zhou J, Tang XC (2002). Huperzine A attenuates apoptosis and mitochondria-dependent caspase-3 in rat cortical neurons. FEBS Lett.

[126] Tang W, Zhang Y, Gao J, Ding X, Gao S (2008). The anti-fatigue effect of 20(R)-ginsenoside Rg3 in mice by intranasally administration. Biol Pharm Bull.

[127] Qian T, Cai Z, Wong RN, Mak NK, Jiang Z-H (2005). In vivo rat metabolism and pharmacokinetic studies of ginsenoside Rg3. J Chromatogr B.

[128] Elliott G, Rechsteiner M (1975). Pyridine nucleotide metabolism in mitotic cells. J Cell Physiol.

[129] Wei CC, Kong YY, Li GQ, Guan YF, Wang P, Miao CY (2017). Nicotinamide mononucleotide attenuates brain injury after intracerebral hemorrhage by activating Nrf2/HO-1 signaling pathway. Sci Rep.

[130] Pandya JD, Readnower RD, Patel SP, Yonutas HM, Pauly JR, Goldstein GA (2014). N-acetylcysteine amide confers neuroprotection, improves bioenergetics and behavioral outcome following TBI. Exp Neurol.

[131] Coles LD, Tuite PJ, Öz G, Mishra UR, Kartha RV, Sullivan KM (2018). Repeated-dose oral N-acetylcysteine in Parkinson’s disease: pharmacokinetics and effect on brain glutathione and oxidative stress. J Clin Pharmacol.

[132] Mischley LK, Lau RC, Shankland EG, Wilbur TK, Padowski JM (2017). Phase IIb study of intranasal glutathione in Parkinson’s disease. J Parkinsons Dis.

[133] Stefanova NA, Muraleva NA, Maksimova KY, Rudnitskaya EA, Kiseleva E, Telegina DV (2016). An antioxidant specifically targeting mitochondria delays progression of Alzheimer’s disease-like pathology. Aging.

[134] Xi Y, Feng D, Tao K, Wang R, Shi Y, Qin H (2018). MitoQ protects dopaminergic neurons in a 6-OHDA induced PD model by enhancing Mfn2-dependent mitochondrial fusion via activation of PGC-1α. Biochim Biophys Acta Mol Basis Dis.

[135] McManus MJ, Murphy MP, Franklin JL (2011). The mitochondria-targeted antioxidant MitoQ prevents loss of spatial memory retention and early neuropathology in a transgenic mouse model of Alzheimer’s disease. J Neurosci.

[136] Haidar MA, Shakkour Z, Barsa C, Tabet M, Mekhjian S, Darwish H (2022). Mitoquinone helps combat the neurological, cognitive, and molecular consequences of open head traumatic brain injury at chronic time point. Biomedicines.

[137] Tabet M, El-Kurdi M, Haidar MA, Nasrallah L, Reslan MA, Shear D (2022). Mitoquinone supplementation alleviates oxidative stress and pathologic outcomes following repetitive mild traumatic brain injury at a chronic time point. Exp Neurol.

[138] Rossman MJ, Santos-Parker JR, Steward CA, Bispham NZ, Cuevas LM, Rosenberg HL (2018). Chronic supplementation with a mitochondrial antioxidant (MitoQ) improves vascular function in healthy older adults. Hypertension.

[139] Speck RM, Foster JJ, Mulhern VA, Burke SV, Sullivan PG, Fleisher LA (2014). Development of a professionalism committee approach to address unprofessional medical staff behavior at an academic medical center. Joint Comm J Quality Patient Safety.

[140] Gupta SC, Patchva S, Koh W, Aggarwal BB (2012). Discovery of curcumin, a component of golden spice, and its miraculous biological activities. Clin Exp Pharmacol Physiol.

[141] Zhu Y-G, Chen X-C, Chen Z-Z, Zeng Y-Q, Shi G-B, Su Y-H (2004). Curcumin protects mitochondria from oxidative damage and attenuates apoptosis in cortical neurons. Acta Pharmacol Sin.

[142] Sorrenti V, Contarini G, Sut S, Dall'Acqua S, Confortin F, Pagetta A (2018). Curcumin prevents acute neuroinflammation and long-term memory impairment induced by systemic lipopolysaccharide in mice. Front Pharmacol.

[143] Ishrat T, Hoda MN, Khan MB, Yousuf S, Ahmad M, Khan MM (2009). Amelioration of cognitive deficits and neurodegeneration by curcumin in rat model of sporadic dementia of Alzheimer’s type (SDAT). Eur Neuropsychopharmacol.

[144] Institute NC (1996). Clinical development plan: curcumin. J Cell Biochem.

[145] Shi Z, Qiu W, Xiao G, Cheng J, Zhang N (2018). Resveratrol attenuates cognitive deficits of traumatic brain injury by activating p38 signaling in the brain. Med Sci Monit.

[146] Liu J, He J, Huang Y, Hu Z (2021). Resveratrol has an overall neuroprotective role in ischemic stroke: a meta-analysis in rodents. Front Pharmacol.

[147] Sawda C, Moussa C, Turner RS (2017). Resveratrol for Alzheimer’s disease. Ann NY Acad Sci.

[148] Yiu EM, Tai G, Peverill RE, Lee KJ, Croft KD, Mori TA (2015). An open-label trial in Friedreich ataxia suggests clinical benefit with high-dose resveratrol, without effect on frataxin levels. J Neurol.

[149] Almeida L, Vaz-da-Silva M, Falcão A, Soares E, Costa R, Loureiro AI (2009). Pharmacokinetic and safety profile of trans-resveratrol in a rising multiple-dose study in healthy volunteers. Mol Nutr Food Res.

[150] Arbo BD, André-Miral C, Nasre-Nasser RG, Schimith LE, Santos MG, Costa-Silva D (2020). Resveratrol derivatives as potential treatments for Alzheimer’s and Parkinson’s disease. Front Aging Neurosci.

[151] Trotta V, Pavan B, Ferraro L, Beggiato S, Traini D, Des Reis LG (2018). Brain targeting of resveratrol by nasal administration of chitosan-coated lipid microparticles. Eur J Pharm Biopharm.

[152] Marx D, Williams G, Birkhoff M (2015). Intranasal drug administration—an attractive delivery route for some drugs drug discovery and development-from molecules to medicine. IntechOpen.

[153] Shamsher E, Sulaimankutty R, Dine K, Luong V, Davis B, Willett K (2020). Intranasal delivery of resveratrol nanoparticles reduces retinal ganglion cell loss in a model of multiple sclerosis. Investig Ophthalmol Vis Sci.

[154] Bao H-j, Qiu H-y, Kuai J-x, Song C-j, Wang S-x, Wang C-q (2016). Apelin-13 as a novel target for intervention in secondary injury after traumatic brain injury. Neural Regen Res.

[155] Chen D, Lee J, Gu X, Wei L, Yu SP (2015). Intranasal delivery of apelin-13 is neuroprotective and promotes angiogenesis after ischemic stroke in mice. ASN Neuro.

[156] de Oliveira MR (2016). Evidence for genistein as a mitochondriotropic molecule. Mitochondrion.

[157] Ahmad N, Ahmad R, Naqvi AA, Alam MA, Ashafaq M, Abdur Rub R (2018). Intranasal delivery of quercetin-loaded mucoadhesive nanoemulsion for treatment of cerebral ischaemia. Artif Cells Nanomed Biotechnol.

[158] Lin ZH, Liu Y, Xue NJ, Zheng R, Yan YQ, Wang ZX (2022). Quercetin protects against MPP(+)/MPTP-induced dopaminergic neuron death in Parkinson’s disease by inhibiting ferroptosis. Oxid Med Cell Longev.

[159] Ghaffari F, Hajizadeh Moghaddam A, Zare M (2018). Neuroprotective effect of quercetin nanocrystal in a 6-hydroxydopamine model of parkinson disease: biochemical and behavioral evidence. Basic Clin Neurosci.

[160] Park DJ, Kang JB, Shah FA, Koh PO (2021). Quercetin attenuates the reduction of parvalbumin in middle cerebral artery occlusion animal model. Lab Anim Res.

[161] Papakyriakopoulou P, Manta K, Kostantini C, Kikionis S, Banella S, Ioannou E (2021). Nasal powders of quercetin-β-cyclodextrin derivatives complexes with mannitol/lecithin microparticles for nose-to-brain delivery: in vitro and ex vivo evaluation. Int J Pharm.

[162] Chen J, Wang J, Wei L, Zhang JH (2019). Therapeutic intranasal delivery for stroke and neurological disorders.

[163] Wang S, Ma F, Huang L, Zhang Y, Peng Y, Xing C (2018). Dl-3-n-butylphthalide (NBP): a promising therapeutic agent for ischemic stroke. CNS Neurol Disord Drug Targets.

[164] Qu M, Zhao J, Zhao Y, Sun J, Liu L, Wei L (2021). Vascular protection and regenerative effects of intranasal DL-3-N-butylphthalide treatment after ischaemic stroke in mice. Stroke Vasc Neurol.

[165] Deutsch J, Rapoport SI, Rosenberger TA (2002). Coenzyme A and short-chain acyl-CoA species in control and ischemic rat brain. Neurochem Res.

[166] Mathew R, Arun P, Madhavarao CN, Moffett JR, Namboodiri MA (2005). Progress toward acetate supplementation therapy for Canavan disease: glyceryl triacetate administration increases acetate, but not N-acetylaspartate, levels in brain. J Pharmacol Exp Ther.

[167] Tefera TW, Wong Y, Barkl-Luke ME, Ngo ST, Thomas NK, McDonald TS (2016). Triheptanoin protects motor neurons and delays the onset of motor symptoms in a mouse model of amyotrophic lateral sclerosis. PLoS ONE.

[168] Mochel F (2017). Triheptanoin for the treatment of brain energy deficit: a 14-year experience. J Neurosci Res.

[169] Kinman RP, Kasumov T, Jobbins KA, Thomas KR, Adams JE, Brunengraber LN (2006). Parenteral and enteral metabolism of anaplerotic triheptanoin in normal rats. Am J Physiol Endocrinol Metab.

[170] Schiffmann R, Mochel F. Triheptanoin diet for adult polyglucosan body disease (apbd) treatment. Google Patents; 2011.

[171] Matern D, Gavrilov DK. Fatty Acid Oxidation Disorders and Epilepsy. Inherited Metabolic Epilepsies. 2012.

[172] Revollo JR, Grimm AA, Imai S (2004). The NAD biosynthesis pathway mediated by nicotinamide phosphoribosyltransferase regulates Sir2 activity in mammalian cells. J Biol Chem.

[173] Diniz YS, Rocha KK, Souza GA, Galhardi CM, Ebaid GM, Rodrigues HG (2006). Effects of N-acetylcysteine on sucrose-rich diet-induced hyperglycaemia, dyslipidemia and oxidative stress in rats. Eur J Pharmacol.

[174] Naoi M, Wu Y, Shamoto-Nagai M, Maruyama W (2019). Mitochondria in neuroprotection by phytochemicals: bioactive polyphenols modulate mitochondrial apoptosis system, function and structure. Int J Mol Sci.

[175] Nicolescu A, Babotă M, Barros L, Rocchetti G, Lucini L, Tanase C (2023). Bioaccessibility and bioactive potential of different phytochemical classes from nutraceuticals and functional foods. Front Nutr.

[176] Md S, Alhakamy NA, Aldawsari HM, Asfour HZ (2019). Neuroprotective and antioxidant effect of naringenin-loaded nanoparticles for nose-to-brain delivery. Brain Sci.

[177] Colombo M, Figueiró F, de Fraga DA, Teixeira HF, Battastini AMO, Koester LS (2018). Kaempferol-loaded mucoadhesive nanoemulsion for intranasal administration reduces glioma growth in vitro. Int J Pharm.

[178] Ahmad N, Ahmad R, Alam MA, Samim M, Iqbal Z, Ahmad FJ (2016). Quantification and evaluation of thymoquinone loaded mucoadhesive nanoemulsion for treatment of cerebral ischemia. Int J Biol Macromol.

[179] Cheung RC, Ng TB, Wong JH, Chan WY (2015). Chitosan: an update on potential biomedical and pharmaceutical applications. Mar Drugs.

[180] Bonferoni MC, Rassu G, Gavini E, Sorrenti M, Catenacci L, Giunchedi P (2020). Nose-to-brain delivery of antioxidants as a potential tool for the therapy of neurological diseases. Pharmaceutics.

[181] Prasuhn J, Davis RL, Kumar KR (2020). Targeting mitochondrial impairment in Parkinson’s disease: challenges and opportunities. Front Cell Dev Biol.

[182] Erdő F, Bors LA, Farkas D, Bajza Á, Gizurarson S (2018). Evaluation of intranasal delivery route of drug administration for brain targeting. Brain Res Bull.

[183] Tamai I, Tsuji AJA (1996). Drug delivery through the blood-brain barrier. Front Cell Dev Biol.

[184] Black IH, McManus J (2009). Pain management in current combat operations. Prehosp Emerg Care.

[185] Christensen K, Rogers E, Green GA, Hamilton DA, Mermelstein F, Liao E (2007). Safety and efficacy of intranasal ketamine for acute postoperative pain. Acute Pain.

[186] Mattarei A, Biasutto L, Marotta E, De Marchi U, Sassi N, Garbisa S (2008). A mitochondriotropic derivative of quercetin: a strategy to increase the effectiveness of polyphenols. ChemBioChem.

[187] Sharma A, Liaw K, Sharma R, Zhang Z, Kannan S, Kannan RM (2018). Targeting mitochondrial dysfunction and oxidative stress in activated microglia using dendrimer-based therapeutics. Theranostics.

[188] Hasan W, Kori RK, Thakre K, Yadav RS, Jat D (2019). Synthesis, characterization and efficacy of mitochondrial targeted delivery of TPP-curcumin in rotenone-induced toxicity. Daru.

[189] Ozsoy Y, Gungor S, Cevher E (2009). Nasal delivery of high molecular weight drugs. Molecules.

[190] Pires A, Fortuna A, Alves G, Falcão A (2009). Intranasal drug delivery: how, why and what for?. J Pharm Pharm Sci.

[191] Wolfe TR, Braude DA (2010). Intranasal medication delivery for children: a brief review and update. Pediatrics.

[192] Chien YW, Chang SF (1987). Intranasal drug delivery for systemic medications. Crit Rev Ther Drug Carrier Syst.

[193] Turner PV, Brabb T, Pekow C, Vasbinder MA (2011). Administration of substances to laboratory animals: routes of administration and factors to consider. J Am Assoc Lab Anim Sci.

[194] Veronesi MC, Alhamami M, Miedema SB, Yun Y, Ruiz-Cardozo M, Vannier MW (2020). Imaging of intranasal drug delivery to the brain. Am J Nucl Med Mol Imaging.

[195] Chenthamara D, Subramaniam S, Ramakrishnan SG, Krishnaswamy S, Essa MM, Lin F-H (2019). Therapeutic efficacy of nanoparticles and routes of administration. Biomaterials Research.

[196] Rajput A, Pingale P, Dhapte-Pawar V (2022). Nasal delivery of neurotherapeutics via nanocarriers: facets, aspects, and prospects. Front Pharmacol.

[197] Wang D, Ren Y, Shao Y, Yu D, Meng L (2017). Facile preparation of doxorubicin-loaded and folic acid-conjugated carbon nanotubes@Poly(N-vinyl pyrrole) for targeted synergistic chemo-photothermal cancer treatment. Bioconjug Chem.

[198] Soligo M, Felsani FM, Da Ros T, Bosi S, Pellizzoni E, Bruni S (2021). Distribution in the brain and possible neuroprotective effects of intranasally delivered multi-walled carbon nanotubes. Nanoscale Adv.

[199] Mohanta D, Patnaik S, Sood S, Das N (2019). Carbon nanotubes: Evaluation of toxicity at biointerfaces. J Pharm Anal.

[200] Bardi G, Nunes A, Gherardini L, Bates K, Al-Jamal KT, Gaillard C (2013). Functionalized carbon nanotubes in the brain: cellular internalization and neuroinflammatory responses. PLoS ONE.

[201] McCully JD, Cowan DB, Emani SM, Pedro J (2017). Mitochondrial transplantation: from animal models to clinical use in humans. Mitochondrion.

[202] Shami GJ, Cheng D, Verhaegh P, Koek G, Wisse E, Braet F (2021). Three-dimensional ultrastructure of giant mitochondria in human non-alcoholic fatty liver disease. Sci Rep.

[203] Alexander JF, Seua AV, Arroyo LD, Ray PR, Wangzhou A, Heiβ-Lückemann L (2021). Nasal administration of mitochondria reverses chemotherapy-induced cognitive deficits. Theranostics.

[204] Liu CS, Chang JC, Kuo SJ, Liu KH, Lin TT, Cheng WL (2014). Delivering healthy mitochondria for the therapy of mitochondrial diseases and beyond. Int J Biochem Cell Biol.

[205] McCully JD, Cowan DB, Pacak CA, Toumpoulis IK, Dayalan H, Levitsky S (2009). Injection of isolated mitochondria during early reperfusion for cardioprotection. Am J Physiol Heart Circ Physiol.

[206] Quintana DS, Guastella AJ, Westlye LT, Andreassen OA (2016). The promise and pitfalls of intranasally administering psychopharmacological agents for the treatment of psychiatric disorders. Mol Psychiatry.

